# Development and Validation of Daping Orthopedics Operative Risk Scoring System for Geriatric Hip Fracture (DOORS‐GHF) for Predicting Postoperative Outcomes in Geriatric Hip Fracture Patients: A Multicenter Retrospective Comparative Study

**DOI:** 10.1111/os.70430

**Published:** 2026-09-27

**Authors:** Zhiwei Gong, Yu Wang, Weiwen Zhu, Jing Zhong, Xiangdong Liu, Runze Jin, Zhaoyuan Zhang, Kesi Liu, Ziming Wang, Xingchen Lu, Yan Xiong

**Affiliations:** ^1^ Department of Orthopaedics Daping Hospital, Army Medical University (Third Military Medical University) Chongqing China; ^2^ Department of Cardiovascular Medicine Xinqiao Hospital, Army Medical University (Third Military Medical University) Chongqing China

**Keywords:** geriatric population, hip fractures, mortality, postoperative complications, risk scoring system

## Abstract

**Objective:**

With China's accelerating population aging, geriatric hip fractures pose a growing clinical challenge. High comorbidity burden and diminished physiological reserve drive elevated postoperative complication and mortality risks in this population. However, most existing systems were developed decades ago, incompatible with modern perioperative advances, and are not tailored for geriatric hip fracture patients, resulting in unsatisfactory predictive specificity. To fill this gap, we developed the Daping Orthopedics Operative Risk Scoring System for Geriatric Hip Fracture (DOORS‐GHF) and evaluated its predictive accuracy and clinical utility.

**Methods:**

We collected 4417 geriatric patients with hip fractures who underwent operative treatment at multicenter hospitals between 2012 and 2025. A total of 1599 patients were finally enrolled in this study. Of these, 1199 cases were included for the development of the scoring system. Using binary logistic regression with preoperative physiological score (PS) and operative severity score (OS) as independent variables, we constructed the DOORS‐GHF model to predict 30‐day postoperative complications and mortality. The remaining 400 cases formed the external validation cohort, in which we compared DOORS‐GHF against the Daping Orthopedic Operative Risk Scoring System for Senile Patient version 3.0 (DORSSSP v3.0) and the Portsmouth Physiological and Operative Severity Score for the Enumeration of Mortality and Morbidity (P‐POSSUM) using AUC, sensitivity, specificity, Hosmer–Lemeshow calibration, Brier score, and decision curve analysis (DCA).

**Results:**

The DOORS‐GHF predictive equations were ln[R/(1 − *R*)] = −5.089 − 0.114 × OS + 0.226 × PS (complications) and ln[Z/(1 − Z)] = −10.308 + 0.655 × OS + 0.201 × PS (mortality). In the validation cohort (*n* = 400), 30‐day mortality was 2.0% (8/400) and complication rate was 6.5% (26/400). DOORS‐GHF outperformed both comparators across all metrics. For complication prediction, it achieved the highest AUC (0.878), acceptable calibration (HL χ^2^ = 11.887, *p* = 0.156), and the lowest Brier score (0.0489). For mortality prediction, it achieved the highest AUC (0.968), good calibration (HL χ^2^ = 6.259, *p* = 0.510), and the lowest Brier score (0.0140). DCA confirmed the broadest clinical net benefit for DOORS‐GHF across threshold ranges of 0–0.65 (complications) and 0–0.40 (mortality).

**Conclusions:**

DOORS‐GHF demonstrated superior discrimination, calibration, accuracy, and clinical utility over DORSSSP v3.0 and P‐POSSUM in geriatric hip fracture patients. As a precise and practical system for assessing the risk of 30‐day postoperative complications and mortality, it holds strong potential to improve perioperative decision‐making and patient counseling in this vulnerable population.

## Graphical abstract

We developed and validated the DOORS‐GHF, a novel dedicated operative risk scoring system for geriatric hip fracture, which outperformed existing scores with superior predictive accuracy and clinical utility.

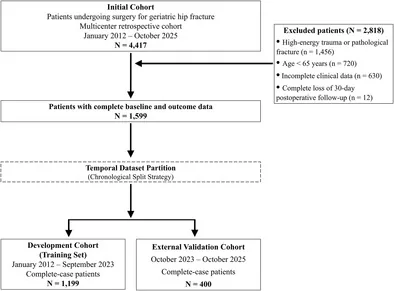

## 1 Introduction

China has entered an aging society, and the geriatric population will continue to impose an increasing burden on families and public healthcare systems [1, 2]. Studies have shown that the global number of hip fracture cases is expected to rise dramatically by 2050, with a particularly significant annual increase in the incidence of hip fractures among geriatric patients [3]. Geriatric hip fractures primarily include femoral neck fractures and intertrochanteric fractures [4], which clinically manifest as hip pain, restricted mobility, and even difficulty standing or walking. It is well established that geriatric hip fractures are associated with high complication rates and mortality. Older adults with hip fractures have a significantly higher mortality rate, especially within the first year, and non‐operative treatment further increases short‐term mortality risk [5, 6]. For older adults with acute hip fractures, surgical intervention remains the mainstay of recommended treatment to reduce mortality and improve functional outcomes [7, 8]. Most orthopedic surgeons advocate early operative intervention as the first‐line treatment for geriatric hip fractures, with total hip arthroplasty (THA) and hemiarthroplasty (HA) typically representing the preferred options for the management of displaced femoral neck fractures and unstable intertrochanteric fractures in geriatric patients [4, 9, 10].

Due to physical frailty, multiple comorbidities, and systemic organ dysfunction, geriatric patients face significantly elevated risks of perioperative mortality and various postoperative complications [11]. Mortality following operative treatment for geriatric hip fractures is substantially higher than that in the general population [12], with approximately 20% of patients aged 65 years and older dying within one year after discharge [13].

As modern nursing models transition from basic care to evidence‐based care [14], there is growing interest in the objective and accurate prediction of postoperative risks using physiological parameters and perioperative clinical indicators during hospitalization, thereby enabling timely adjustments to clinical and nursing strategies [15, 16, 17]. Operative risk scoring systems developed based on clinical indicators quantify operative risk on a percentage scale, allowing clinicians to assess risk more intuitively, enhance perioperative vigilance, and make clinical decisions earlier [18]. Such scoring systems play a crucial role in risk stratification, guiding perioperative management, and improving patient prognosis, which is particularly important for geriatric hip fracture patients with complex conditions.

Currently, a variety of operative risk scoring systems have been developed for clinical application. Among them, the Portsmouth Physiological and Operative Severity Score for the Enumeration of Mortality and Morbidity (P‐POSSUM) is commonly used internationally [19, 20], while the Daping Orthopedic Operative Risk Scoring System for Senile Patients (DORSSSP) is widely applied in China [21, 22]. However, many studies have indicated that the P‐POSSUM system overestimates operative risk in geriatric hip fracture patients, leading to unnecessary clinical interventions [23, 24]. Nevertheless, P‐POSSUM system was developed based on foreign populations and may not be fully applicable to Chinese geriatric patients. Our team previously established the DORSSSP system in 2013, which was developed within China's orthopedic medical framework using data from domestic populations and excludes certain indicators from the P‐POSSUM system [25]. However, with continuous advances in medical technology and improvements in perioperative management, the DORSSSP system also overestimates operative risk in current clinical practice [26]. In addition, neither scoring system comprehensively assesses the risk of comorbidities in geriatric patients, which may be an important factor affecting postoperative outcomes [27, 28], and the weighting of surgical‐related factors may not be appropriately calibrated for this specific population, as the surgical severity weighting designed for general surgical patients may not fully match the characteristics of geriatric hip fracture surgery [29, 30]. Recent advances in medical care have significantly altered the clinical profiles of geriatric hip fracture patients, and these scoring systems, developed in a different clinical era, may no longer be appropriately calibrated for risk stratification in the current Chinese geriatric population [31, 32].

To address these limitations, we developed the Daping Orthopedics Operative Risk Scoring System for Geriatric Hip Fracture (DOORS‐GHF), a novel risk model specifically calibrated for the Chinese geriatric population undergoing hip fracture surgery. The model integrates the Age‐Adjusted Charlson Comorbidity Index (aCCI) to better quantify comorbidity burden and refines the weighting of operative severity factors. This study therefore aimed to validate the accuracy and clinical applicability of this new scoring system in predicting 30‐day postoperative complications and mortality.

## 2 Materials and Methods

### 2.1 Data Source and Variables

This retrospective multicenter study was conducted on 4417 geriatric patients with hip fractures who underwent operative treatment at three tertiary hospitals in China between January 2012 and October 2025: Chongqing Daping Hospital and Xinqiao Hospital (both affiliated with the Army Medical University; data from the two hospitals were analyzed jointly) and the First Affiliated Hospital of Chongqing Medical University.

### 2.2 The Inclusion Criteria

(i) patients aged 65 years or older, (ii) low‐energy or fragility hip fractures in accordance with International Classification of Diseases coding criteria, including femoral neck fractures: shortening of the affected limb, external rotation deformity, severe hip pain aggravated by motion, with or without hip swelling and ecchymosis, or fracture lines visible on anteroposterior and lateral pelvic radiographs, or three‐dimensional computed tomography (CT) confirming femoral neck fracture. Intertrochanteric fracture: clear history of trauma, longitudinal percussion pain over the hip, swelling with ecchymosis, impaired hip function, and intertrochanteric fracture confirmed by anteroposterior and lateral pelvic radiographs or CT scans, (iii) patients who have been evaluated as having no operative contraindications on preoperative evaluation and underwent hip fracture surgery, and (iv) patients with complete clinical data available. The exclusion criteria: (i) high‐energy trauma (e.g., fractures caused by traffic accidents) or pathologic fractures secondary to malignant tumors, (ii) incomplete clinical data, (iii) patients lost the follow‐up results completely.

We employed temporal splitting: patients treated between January 2012 and September 2023 constituted the development cohort (*n* = 1199; 341 males, 858 females; median age, 80 years [IQR: 73–85]), and those treated between October 2023 and October 2025 formed the independent external validation cohort (*n* = 400; 119 males, 281 females; median age, 77 years [IQR: 71–83]), as shown in Figure 1. DOORS‐GHF was developed using the development cohort, in which 125 patients (10.4%) experienced surgery‐related complications within one month postoperatively and 22 (1.8%) died. The validation cohort served to benchmark DOORS‐GHF against two existing scoring systems. Within 30 days postoperatively, 26 patients developed complications and 8 died. Through descriptive statistical analysis, we summarized the baseline characteristics of the study population: for all variables, the total numbers and cases of survival/death in different categories were counted. Categorical variables were described as number (proportion, n%), with binary variables analyzed using Chi‐square test if minimum expected frequency ≥ 5 or Fisher's exact test if < 5, multinomial variables used Chi‐square test. Continuous variables underwent normality testing: normally distributed data were presented as mean ± standard deviation (x ± s) with intergroup comparisons by independent samples *t*‐test, non‐normally distributed data were expressed as median (interquartile range) [M(IQR)] and compared using Mann–Whitney U test.

**FIGURE 1:**
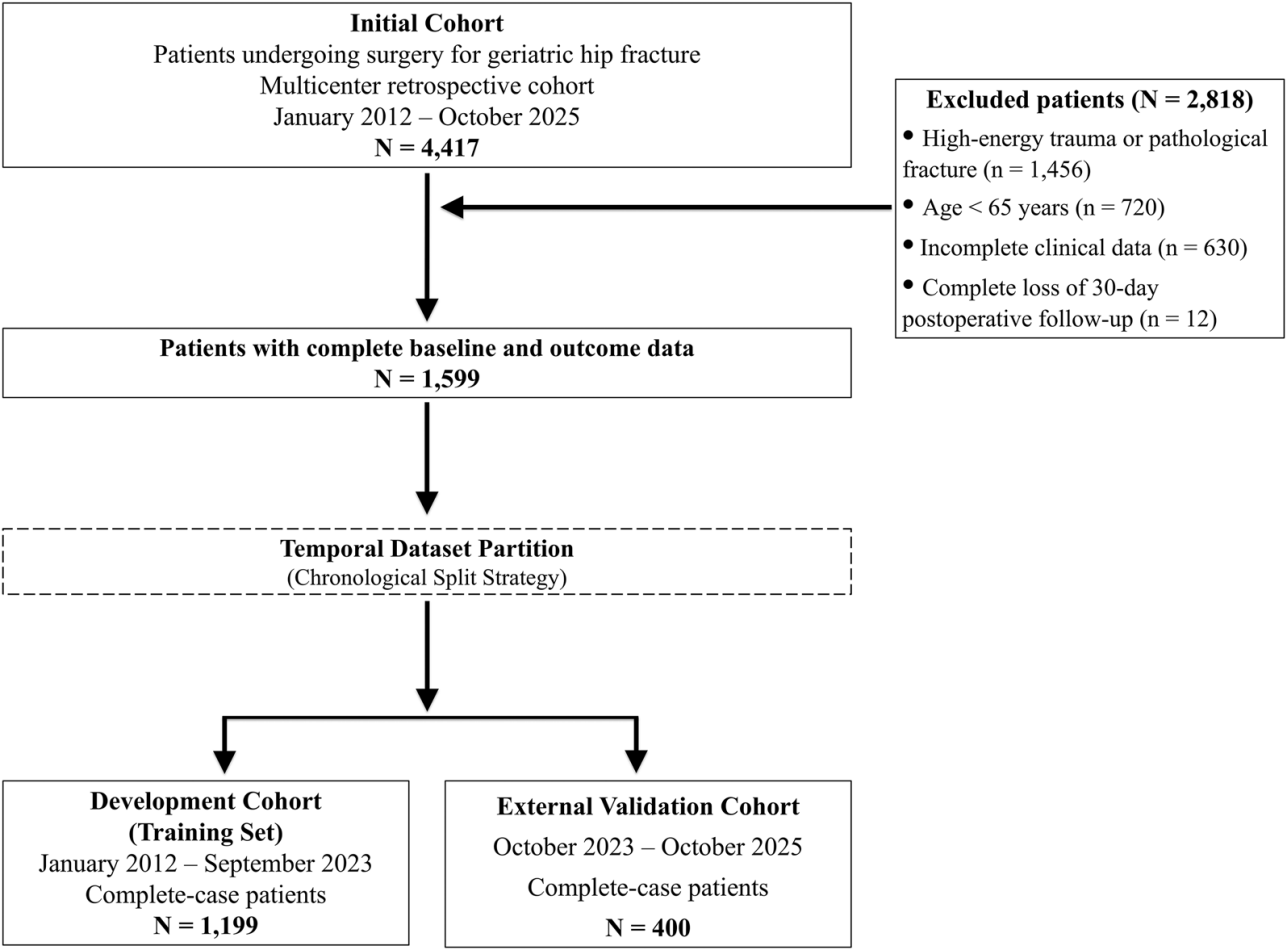
Flowchart of population screening in training and validation cohort.

The ethical approval for this study (Ethical Approval No. 331) was provided by the Medical Research Ethics Review Committee of the PLA Army Medical Center, Chongqing, China on 20 November 2024. All authors had access to the study data, reviewed and revised the final manuscript, and approved the decision to submit the manuscript for publication.

### 2.3 Outcome Measures

Thirty‐day postoperative complications mainly include respiratory complications, cardiac complications, urinary complications, digestive complications, pressure injuries, neurological complications, and deep vein thrombosis (DVT) of the lower extremities. The diagnostic criteria are as follows:

Respiratory complications: pulmonary infection, pulmonary embolism, and respiratory failure, diagnosed by clinical symptoms (fever, cough, expectoration, dyspnea) combined with chest X‐ray, chest CT, or pulmonary function tests. Cardiac complications: myocardial infarction, heart failure, and arrhythmia, confirmed by electrocardiogram and echocardiography. Urinary complications: urinary tract infection and urinary retention, diagnosed by urinalysis combined with clinical manifestations. Digestive complications: mainly gastrointestinal bleeding and diarrhea, confirmed by stool routine examination and clinical symptoms. Neurological complications: intracerebral hemorrhage and cerebral infarction, confirmed by CT imaging combined with clinical manifestations.

### 2.4 Risk Assessment System

The DOORS‐GHF system was developed based on our previously established DORSSSP v3.0 scoring system, with systematic optimization tailored specifically for geriatric patients with hip fractures. By incorporating multicenter clinical data from geriatric hip fracture patients who underwent operative treatment in recent years and re‐optimizing the prediction formulas for both complication incidence and mortality, DOORS‐GHF aims to more accurately predict postoperative risk rates specific to this patient population. The preoperative physiological parameter scores (PS) comprise ten variables: Age, Cardiac Function Classification, White Blood Cell count (WBC), Hemoglobin (Hb), Systolic Blood Pressure, Blood Urea Nitrogen (BUN), Diabetes Mellitus Classification, Electrocardiogram (ECG), Fracture Type, and Age‐adjusted Charlson Comorbidity Index (aCCI). Each variable is graded and assigned a score according to established classification criteria. Specifically, cardiac function is classified into four classes (I–IV) based on exercise tolerance and breath‐holding test performance, consistent with the revised criteria of the New York Heart Association (NYHA) [33] (Table S4). Systolic blood pressure is categorized according to the Chinese Guidelines for the Prevention and Treatment of Hypertension (2024 Revised Edition). Diabetes mellitus is stratified into four health status grades based on comorbidity burden, cognitive function, and activities of daily living impairment, in accordance with the Guideline for the Management of Diabetes Mellitus in the Elderly in China (2024 edition) [34] (Table S3). The aCCI is calculated as the sum of the comorbidity weight score and the age adjustment score, following the classic Charlson Comorbidity Index framework and its validated age‐adjusted version [35, 36] (Table S7). Notably, unlike DORSSSP v3.0 and P‐POSSUM, which incorporate pulmonary function classification graded by the degree of dyspnea during daily activities according to the criteria established by Fletcher et al. [37] (Table S5). DOORS‐GHF deliberately excludes this parameter and instead incorporates fracture type and aCCI to better capture the population‐specific risk profile of geriatric hip fracture patients. The specific scoring items and their assigned scores for the preoperative physiological parameters are shown in Table 2. The operative risk parameter scores (OS) include three variables: type of surgery, operative time, and intraoperative blood loss. Surgical procedures are classified into minor, moderate, major, and extra‐major categories based on surgical risk, procedural complexity, and technical difficulty, in accordance with the Measures for the Administration of Surgical Grading in Medical Institutions issued by the National Health Commission of China (2022) [38] (Table S6). Intraoperative blood loss is estimated based on the surgical procedure type (hemiarthroplasty or total hip arthroplasty) and age group, using the mean blood loss of the corresponding subgroup derived from the development cohort as the estimated value (Table S8). The specific scoring items and their assigned scores for the operative risk parameters are shown in Table 1. The PS is the sum of the subscores of the ten physiological items (Table 2), and the OS is the sum of the subscores of the three operative items (Table 1); each item is assigned 0, 1, 2, 4, or 8 points according to its classification rules (Tables [Link], [Link]). The item‐level scoring schemes were fixed a priori on the basis of the established DORSSSP v3.0 criteria and were not derived from univariate screening or re‐selected from the current data; only the regression coefficients linking PS and OS to the outcomes were estimated from the development cohort. This study employed a binomial logistic regression model to develop the brand‐new predictive models. The core principle involves mapping unbounded linear predictions to probability values between 0 and 1 through a logit transformation, thereby quantifying the association between independent variables and the probability of clinical outcomes. Let P represent the probability of an event occurrence (P denotes 1‐month postoperative mortality in the mortality model, and indicates postoperative complication incidence in the complication model, with 0 ≤ *p* ≤ 1). The logit transformation is defined as the natural logarithm of the odds ratio (the ratio of event occurrence probability to non‐occurrence probability), expressed as:

$$\text{logit}\left(P\right)=\ln\left[P/\left(1-P\right)\right]$$

where ln(·) represents the natural logarithm, and *P*/(1–*P*) denotes the odds ratio with a range from 0 to +∞. Following the logit transformation, the probability space (0–1) is mapped to an unbounded real number space (−∞ to +∞), satisfying the assumptions of linear modeling. The general form of the logistic regression linear prediction equation is:

$$\begin{array}{c} \ln\left[P/\left(1-P\right)\right]=\beta_{0}+\beta_{1}\times \mathrm{OS}+\beta_{2}\times \mathrm{PS} \end{array}$$

Here, *β*_0_ is the intercept term, *β*_1_ and *β*_2_ represent regression coefficients for the independent variables OS and PS, respectively. All parameters are fitted using the maximum likelihood estimation (MLE) method. The probability of event occurrence can be directly calculated using the inverse logit transformation:

$$\begin{array}{c} P=\exp\left(\beta_{0}+\beta_{1}\times \mathrm{OS}+\beta_{2}\times \mathrm{PS}\right)/\left[1+\exp\left(\beta_{0}+\beta_{1}\times \mathrm{OS}+\beta_{2}\times \mathrm{PS}\right)\right] \end{array}$$

where exp(·) denotes the natural exponential function. This operation converts unbounded linear predictions into positive odds ratios, with the denominator (1 + odds ratio) ensuring normalization. This mathematical formulation guarantees that *P* remains within the 0–1 interval, satisfying fundamental probability definitions.

**TABLE 1:** DOORS‐GHF operative severity score (OS) assignment.

Item	1	2	4	8
Type of surgery	Small	Medium	Large	—
Operation time, (min)	≤ 60	60 < time ≤ 120	> 120	—
Bleeding volume, (mL)	< 400	400–800	—	> 800

**TABLE 2:** DOORS‐GHF preoperative physiological factor assignment score.

Item	0	1	2	4	8
Age (years)	—	60–69	70–79	80–89	≥ 90
Cardiac function class	—	Class 1	Class 2	Class 3	Class 4
White blood cells, (WBC) (10^9^/L)	—	4 ≤ WBC ≤ 10	WBC < 4; WBC > 10	—	—
Hemoglobin, (HB) (g/L)	≥ 130	110 ≤ Hb < 130	90 ≤ Hb < 110	60 ≤ Hb < 90	< 60
Systolic blood pressure, (mmHg)	< 140	140–159	160–179	—	≥ 180
Blood urea nitrogen, (BUN), (mmol/L)	< 7.5	—	7.5 ≤ BUN ≤ 10	10 < BUN ≤ 15	> 15
Diabetes mellitus class	—	Class 1	Class 2	Class 3	Class 4
Electrocardiogram, (ECG)	—	Normal ECG	Other abnormal ECG	—	—
Fracture type	—	Femoral neck fracture	Intertrochanteric fracture	—	—
Age‐adjusted charlson comorbidity index	—	< 3	3 ≤ Value ≤ 6	—	> 6

The binary logistic regression model was constructed using R software (version 4.5.0) with the glm() function, with the actual outcomes of complications or mortality within 1 month postoperatively defined as the dependent variable and OS/PS designated as the independent variable:

$$\mathrm{glm}\left(\text{outcome}∼\mathrm{OS}+\mathrm{PS},\text{family}=\text{binomial}\right)$$

Following model fitting, parameter estimates were extracted using the summary() function, including the intercept term *β*_0_, OS regression coefficient *β*_1_, and PS regression coefficient *β*_2_. These parameters were incorporated into the logistic regression linear predictor equation to derive the core calculation formula for complications or mortality risk prediction.

The DORSSSP system incorporates preoperative physiological parameter scores and operative risk parameter scores, with the total score representing the operative risk (ranging from 12 to 120 points, higher scores indicate greater risk) [11]. The DORSSSP v3.0 builds upon this foundation by expanding the analyzed case volume, systematically analyzing risk parameters through integrated statistical methodologies, reassigning weights, and implementing stratified classifications based on patients' pre‐treatment conditions—including preoperative physiological and operative risk parameter score. Two clinicians independently evaluate patients and submit their assessments to a third clinician for final review [27]. The DORSSSP v3.0 score prediction equation is: ln[Z/(1–Z)] = −4.643 + 0.1428 × PS + 0.1853 × OS, ln[R/(1 − R)] = −6.999 + 0.1299 × PS + 0.2562 × OS, where R is the postoperative complication, Z represents the postoperative mortality.

The POSSUM system includes preoperative physiological parameter scores and operative risk parameter scores [15, 29]. Preoperative physiological parameters encompass age, cardiac function classification, pulmonary function classification, hemoglobin, blood pressure, renal function, Glasgow coma scale, heart rate, serum sodium, serum potassium, WBC, and electrocardiogram. Operative risk parameters include operative magnitude, duration, intraoperative blood loss, frequency, and timing. The P‐POSSUM system builds upon this by increasing the number of analyzed cases, integrating statistical methods to reanalyze risk parameters, reweighting the factors, and reclassifying patients based on preoperative characteristics. The mortality prediction formula was further refined to improve the accuracy of predicted mortality rates compared to actual outcomes. The P‐POSSUM score prediction equation is: ln[Z/(1 − Z)] = −9.065 + 0.1692 × PS + 0.1550 × OS, ln[R/(1 − R)] = −5.91 + 0.16 × PS + 0.19 × OS, R is the postoperative complication, Z represents the postoperative mortality.

The DOORS‐GHF, DORSSSP v3.0, and P‐POSSUM systems exhibit variations in the specific parameters they employ for risk assessment. (Table 3).

**TABLE 3:** Comparison of evaluation factors among DOORS‐GHF, DORSSSP v3.0, and P‐POSSUM.

Evaluation factors	DOORS‐GHF	DORSSSP v3.0	P‐POSSUM
Age (years)	✓	✓	✓
Cardiac function classification	✓	✓	✓
Pulmonary function classification		✓	✓
Hemoglobin (Hb)	✓	✓	✓
White blood cell (WBC)	✓		✓
Systolic pressure	✓	✓	✓
Blood urea NITROGEN (BUN)	✓	✓	✓
Pulse			✓
Glasgow coma scale (GCS)		✓	✓
Classification of diabetes	✓	✓	
Electrocardiogram (ECG)	✓		✓
Fracture type (femoral neck fractures/intertrochanteric fracture)	✓		
Age‐adjusted Charlson comorbidity index (aCCI)	✓		
NA+			✓
K+			✓
Major/Minor surgery classification	✓	✓	✓
Surgical duration	✓	✓	
Intraoperative blood loss	✓	✓	✓
Number of surgeries		✓	✓
Surgical timing classification		✓	✓
Wound infection types			✓
Malignancy grade			✓

### 2.5 Discrimination

Researchers used receiver operating characteristic (ROC) curves to evaluate the discrimination of the prediction systems, with the area under the curve (AUC) as the primary metric. The AUC ranges from 0.5 to 1.0, where an AUC ≥ 0.80 indicates excellent discrimination, 0.70–0.79 indicates good discrimination, and 0.50–0.69 indicates poor discrimination. Additionally, the Youden index maximization method was employed to determine the optimal cutoff value for each system, and the sensitivity and specificity were calculated at the corresponding threshold value to evaluate the model's ability to identify true positive cases and true negative cases. The violin plots, visually presenting the distribution characteristics of predicted complication incidence and mortality rates from DOORS‐GHF, DORSSSP v3.0, and P‐POSSUM. The width of the violin plot represents data density, the thick dashed line in the middle indicates the median, the thin dashed lines above and below represent quartiles (Q1/Q3), and the fine lines extending at both ends indicate outliers. This plot intuitively displays the central tendency, dispersion, and distributional shape of the predictive values from each system.

### 2.6 Calibration

Calibration is a measure of the agreement between predicted and observed outcomes. We assessed the calibration of the prediction systems using the HL test [31] and the Brier score [39]. The HL test stratifies patients into 10 groups based on predicted probability of death and compares the predicted mortality rate with the actual observed mortality rate in each group. Results are reported as chi‐square (*χ* ^2^), degrees of freedom (DF), and *p* value. A *p* > 0.05 indicates no significant difference between predicted probabilities and observed frequencies, suggesting good model fit.

The Brier score was used to quantitatively evaluate the overall predictive accuracy of the three systems. It is calculated using the formula:

$$\mathrm{BS}=\frac{1}{n}\sum_{i=1}^{n}{\left(p_{i}-y_{i}\right)}^{2}$$

where *p*_i_ is the predicted probability of death for the i‐th patient, and *y*_i_ is the observed outcome (Death = 1, Survival = 0 or Complication occurred = 1, No complication occurred = 0). The Brier score ranges from 0 to 1, with lower values indicating higher overall predictive accuracy.

### 2.7 Clinical Utility

To evaluate the clinical net benefit and practical utility of the three prediction systems for postoperative complication incidence and mortality in patients with hip fractures, we performed decision curve analysis (DCA). DCA is a method that assesses the clinical value of a predictive model by quantifying and comparing its “net benefit” across a range of threshold probabilities, thereby integrating predictive performance with the clinical consequences of decisions—specifically, the benefits of true positives versus the harms of false positives. Net benefit accounts for both the gains from correctly identifying high‐risk patients who benefit from intervention and the losses from unnecessary interventions in low‐risk individuals.

We explored a threshold probability range from 0 to 1, which encompasses the clinically relevant decision points for intervention in this patient population. The threshold probability (plotted on the x‐axis) represents the minimum predicted risk at which a clinical intervention would be triggered. In this study, the target behavior was the decision to escalate perioperative care—for example, preoperative intensive care consultation, invasive hemodynamic monitoring, or enhanced postoperative surveillance—for patients whose predicted risk of 30‐day postoperative complications or mortality was equal to or greater than the threshold; below the threshold, no intervention was undertaken. For each threshold probability, the net benefit was calculated using the following formula:

$$\mathrm{Net}\;\text{Benefit}=\frac{\text{True Positives}}{N}-\frac{\text{False Positives}}{N}\times \frac{\text{Threshold Probability}}{1-\text{Threshold Probability}}$$

where *N* is the total sample size. The term serves as a weighting factor that reflects the relative harm of a false‐positive decision compared with a false‐negative one. In this study, we assumed equal clinical consequences for over‐treatment and under‐treatment (i.e., one unnecessary intervention is as harmful as one missed intervention), resulting in a weight of 1. Two reference strategies were included for comparison:

1. The treat‐all strategy (intervening in all patients, yielding 100% sensitivity but 100% false‐positive rate), and.
2. The treat none strategy (intervening in no patients, yielding 0% false positives but 100% false negatives).

The treat‐all strategy produces a straight line with a negative slope, the treat‐none strategy has a constant net benefit of zero (i.e., aligned with the x‐axis). All DCA analyses were conducted using the DCA curve module of the SPSSAU online statistical platform (version V25.0, https://spssau.com). In the analysis, the observed outcome (Death = 1, Survival = 0 or Complication occurred = 1, No complication occurred = 0) was specified as the status variable, and the predicted probabilities from each of the three systems were entered separately as predictor variables. The threshold probability range for evaluation was set from 0 to 1. Bootstrap resampling (1000 iterations) was performed to estimate 95% confidence bands for all DCA curves, confirming the stability of the net benefit estimates across clinically relevant threshold ranges.

### 2.8 Statistical Analysis

All statistical analyses strictly followed biostatistical guidelines, with data processing conducted using R (v4.5.0) and SPSS (v27.0.1). Significance was defined as *α* = 0.05 (two‐tailed), with *p* < 0.05 indicating statistical significance. Categorical variables were described as *n* (%) and analyzed via chi‐square tests (minimum expected count ≥ 5) or Fisher's exact tests (< 5), while continuous variables underwent normality testing: normally distributed data were expressed as mean ± SD and compared using independent *t*‐tests, and non‐normal data as median (interquartile range [M (IQR)]) with Mann–Whitney U tests. Predictive formulas for mortality and complication rates were derived in R (v4.5.0) using clinically relevant indicators. Discrimination was evaluated via ROC curves and AUC with 95% CIs (R v4.5.0), with validation set AUC stability confirmed in SPSS (v27.0.1). Calibration was assessed using the Hosmer‐Lemeshow test (*χ* ^2^ distribution with degrees of freedom = groups‐2), where *p* > 0.05 indicated optimal calibration. Brier scores (range 0–1) quantified prediction accuracy (lower values = higher precision) in R (v4.5.0). Clinical utility was determined through DCA using SPSSAU, calculating net benefits across threshold probabilities to evaluate clinical decision‐making applicability.

## 3 Results

### 3.1 Baseline Characteristics

The study population was divided into development and validation cohorts according to the study design. The development cohort included 1199 geriatric hip fracture patients for model development and training, while the validation cohort comprised 400 independent patients for external validation. Demographic and clinical characteristics are summarized in Tables 4 and 5.

**TABLE 4:** Demographic characteristics of the development cohort.

Parameters	Total (*n* = 1199)	Complication results		Statistics	*p* value	Death results		Statistics	*p* value
		No (*n* = 1074)	Yes (*n* = 125)			No (*n* = 1177)	Yes (*n* = 22)		
Gender, *n* (%)				χ^2^ = 0.553	0.457			χ2 = 0.126	0.723
Male	341 (28.440)	309 (28.771)	32 (25.600)			334 (28.377)	7 (31.818)		
Female	858 (71.560)	765 (71.229)	93 (74.400)			843 (71.623)	15 (68.182)		
Age, (year) Mean ± SD	80.000 (73.000, 85.000)	80.000 (73.000, 85.000)	81.000 (73.000, 87.000)	Z = −1.085	0.278	78.671 ± 9.351	84.318 ± 7.448	Z = −3.244	0.001
Cardiac function Classification, *n* (%)				Z = −0.559	0.576			Z = −2.431	0.015
I	576 (48.040)	514 (47.858)	62 (49.600)			572 (48.598)	4 (18.182)		
II	346 (28.857)	321 (29.889)	25 (20.000)			335 (28.462)	11 (50.000)		
III	277 (23.103)	239 (22.253)	38 (30.400)			270 (22.940)	7 (31.818)		
WBC, M (Q_1_, Q_3_)	7.580 (6.113, 9.290)	7.620 (6.140, 9.290)	7.070 (5.920, 9.270)	Z = 1.598	0.110	7.570 (6.110, 9.290)	8.230 (6.397, 9.643)	Z = −0.4201	0.675
HB, Mean ± SD	115.490 ± 37.608	115.531 ± 39.289	115.136 ± 17.511	*t* = 0.111	0.912	115.601 ± 37.872	109.545 ± 18.092	*t* = 0.748	0.455
BUN, M (Q_1_, Q_3_)	6.330 (5.012, 8.170)	6.330 (5.010, 8.200)	6.300 (5.070, 7.840)	Z = 0.674	0.500	6.325 (5.010, 8.133)	7.745 (5.140, 10.865)	Z = −1.315	0.188
Systolic pressure (mmHg), Mean ± SD	142.403 ± 48.894	142.428 ± 51.077	142.192 ± 22.873	*t* = 0.051	0.959	142.347 ± 49.234	145.409 ± 25.021	*t* = −0.291	0.771
Fracture type (Garden/Evans), *n* (%)				χ^2^ = 11.446	0.178			χ2 = 16.346	0.038
Garden	830 (69.224)	743 (89.518)	87 (10.482)			822 (99.036)	8 (0.964)		
Evans	369 (30.776)	331 (89.702)	38 (10.298)			355 (96.206)	14 (3.794)		
Hypertension, *n* (%)				χ^2^ = 0.457	0.499			χ2 = 1.438	0.230
No	642 (53.545)	571 (53.166)	71 (56.800)			633 (53.781)	9 (40.909)		
Yes	557 (46.455)	503 (46.834)	54 (43.200)			544 (46.219)	13 (59.091)		
Cancer, *n* (%)					0.645			χ2 = 0.005	0.945
No	1148 (95.746)	1029 (95.81)	119 (95.200)			1127 (95.752)	21 (95.455)		
Yes	51 (4.254)	45 (4.190)	6 (4.800)			50 (4.248)	1 (4.545)		
Coronary heart disease, *n* (%)				χ^2^ = 0.253	0.615			χ2 = 0.371	0.542
No	834 (69.558)	750 (69.832)	84 (67.200)			820 (69.669)	14 (63.636)		
Yes	365 (30.442)	324 (30.168)	41 (32.800)			357 (30.331)	8 (36.364)		
Cerebrovascular disease, *n* (%)				χ^2^ = 0.070	0.791			χ2 = 11.681	0.001
No	1002 (83.570)	896 (83.426)	106 (84.800)			990 (84.112)	12 (54.545)		
Yes	197 (16.430)	178 (16.574)	19 (15.200)			187 (15.888)	10 (45.455)		
Nervous system disease, *n* (%)				χ^2^ = 0.721	0.396			χ2 = 0.002	0.967
No	1093 (91.159)	976 (90.875)	117 (93.600)			1073 (91.164)	20 (90.909)		
Yes	106 (8.841)	98 (9.125)	8 (6.400)			104 (8.836)	2 (9.091)		
Respiratory system disease, *n* (%)				χ^2^ = 1.066	0.302			χ2 = 14.596	< 0.001
No	976 (81.401)	879 (81.844)	97 (77.600)			965 (81.988)	11 (50.000)		
Yes	223 (18.599)	195 (18.156)	28 (22.400)			212 (18.012)	11 (50.000)		
Digestive system disease, *n* (%)				χ^2^ = 5.215	0.022			χ2 = 1.757	0.185
No	1081 (90.158)	976 (90.875)	105 (84.000)			1063 (90.314)	18 (81.818)		
Yes	118 (9.842)	98 (9.125)	20 (16.000)			114 (9.686)	4 (18.182)		
Hepatic/Irenal insufficiency, *n* (%)					0.037			χ2 = 2.769	0.096
No	1061 (88.490)	958 (89.199)	103 (82.400)			1044 (88.700)	17 (77.273)		
Yes	138 (11.510)	116 (10.801)	22 (17.600)			133 (11.300)	5 (22.727)		
Diabetes, *n* (%)				χ^2^ = 0.004	0.951			χ2 = 0.229	0.632
No	923 (76.981)	826 (76.909)	97 (77.600)			907 (77.060)	16 (72.727)		
Yes	276 (23.019)	248 (23.091)	28 (22.400)			270 (22.940)	6 (27.273)		
Ectopic rhythm, *n* (%)				χ^2^ = 0.374	0.541			χ2 = 0.050	0.824
No	1092 (91.076)	980 (91.248)	112 (89.600)			1074 (91.249)	18 (81.818)		
Yes	107 (8.924)	94 (8.752)	13 (10.400)			103 (8.751)	4 (18.182)		
Tachyarrhythmian (%)				χ^2^ = 1.493	0.222			χ2 = 0.017	0.898
No	1099 (91.660)	988 (91.993)	111 (88.800)			1079 (91.674)	20 (90.909)		
Yes	100 (8.340)	86 (8.007)	14 (11.200)			98 (8.326)	2 (9.091)		
Conduction disorder, *n* (%)				χ^2^ = 4.745	0.314			χ2 = 0.383	0.996
No	985 (82.152)	888 (82.682)	97 (77.600)			967 (82.158)	18 (81.818)		
Atrial premature beats	201 (16.764)	173 (16.108)	28 (22.400)			199 (16.907)	2 (9.091)		
Supraventricular premature contractions	2 (0.167)	2 (0.186)	0 (0.000)			2 (0.170)	0 (0.000)		
Ventricular premature contractions	11 (0.917)	11 (1.024)	0 (0.000)			9 (0.765)	2 (9.091)		
Premature beat, *n* (%)				χ^2^ = 5.575	0.350			χ2 = 3.001	0.558
No	1042 (86.906)	937 (87.244)	105 (84.000)			1024 (87.001)	18 (81.818)		
Yes	157 (13.094)	137 (12.756)	20 (16.000)			153 (12.999)	4 (18.182)		
ECG band change, *n* (%)				χ^2^ = 0.166	0.921			χ2 = 0.127	0.938
No	960 (80.067)	859 (79.981)	101 (80.800)			943 (80.119)	17 (77.273)		
Yes	239 (19.933)	215 (20.019)	24 (19.200)			234 (19.881)	5 (22.727)		
Operation time (min), M (Q_1_, Q_3_)	85.000 (70.000, 105.000)	85.000 (70.000, 105.750)	80.000 (65.000, 101.000)	Z = 1.333	0.183	85.000 (70.000, 105.000)	76.000 (62.000, 93.750)	Z = 1.461	0.144
Bleeding volume (mL), *n* (%)				Z = −2.214	0.027			Z = 0.116	0.908
50	8 (0.667)	8 (0.745)	0 (0.000)			8 (0.680)	0 (0.000)		
100	41 (3.420)	35 (3.259)	6 (4.800)			40 (3.398)	1 (4.545)		
150	68 (5.671)	65 (6.052)	3 (2.400)			67 (5.692)	1 (4.545)		
200	188 (15.680)	173 (16.108)	15 (12.000)			187 (15.888)	1 (4.545)		
250	90 (7.506)	81 (7.542)	9 (7.200)			88 (7.477)	2 (9.091)		
300	215 (17.932)	199 (18.529)	16 (12.800)			210 (17.842)	5 (22.727)		
350	112 (9.341)	101 (9.404)	11 (8.800)			105 (8.921)	7 (31.818)		
400	172 (14.345)	143 (13.315)	29 (23.200)			171 (14.528)	1 (4.545)		
450	98 (8.173)	86 (8.007)	12 (9.600)			95 (8.071)	3 (13.636)		
500	74 (6.172)	68 (6.331)	6 (4.800)			74 (6.287)	0 (0.000)		
550	36 (3.003)	25 (2.328)	11 (8.800)			36 (3.059)	0 (0.000)		
600	39 (3.253)	36 (3.352)	3 (2.400)			39 (3.314)	0 (0.000)		
650	57 (4.754)	53 (4.935)	4 (3.200)			56 (4.758)	1 (4.545)		
700	1 (0.083)	1 (0.093)	0 (0.000)			1 (0.085)	0 (0.000)		

**TABLE 5:** Demographic characteristics of the external validation cohort.

Parameters	Total (*n* = 400)	Complication results		Statistics	*p* value	Death outcomes		Statistics	*p* value
		No (*n* = 374)	Yes (*n* = 26)			No (*n* = 392)	Yes (*n* = 8)		
Gender, *n* (%)				χ2 = 1.4721	0.225			χ2 = 0.088	0.767
Male	119 (29.750)	114 (30.481)	5 (19.231)			117 (29.847)	2 (25.000)		
Female	281 (70.250)	260 (69.519)	21 (80.769)			275 (70.153)	6 (75.000)		
Age, (year) Mean ± SD	77.000 (71.000, 83.000)	77.000 (71.000, 82.000)	83.000 (80.250, 86.500)	Z = −4.595	< 0.001	77.000 (71.000, 83.000)	77.500 (75.250, 80.250)	Z = −0.346	0.729
Cardiac function Classification, *n* (%)				Z = −0.911	0.362			Z = −0.554	0.579
I	115 (28.750)	109 (29.144)	6 (23.077)			114 (29.082)	1 (12.500)		
II	128 (32.000)	122 (32.620)	6 (23.077)			125 (31.888)	3 (37.500)		
III	103 (25.750)	91 (24.332)	12 (46.154)			99 (25.255)	4 (50.000)		
IV	54 (13.500)	52 (13.904)	2 (7.692)			54 (13.776)	0 (0.000)		
WBC, M (Q_1_, Q_3_)	7.480 (6.137, 9.535)	7.465 (6.122, 9.518)	7.690 (6.545, 9.430)	Z = −0.275	0.783	7.525 (6.148, 9.580)	6.250 (5.268, 6.900)	Z = 1.832	0.067
HB, Mean ± SD	123.578 ± 57.293	124.570 ± 58.930	109.308 ± 18.695	*t* = 1.315	0.189	124.028 ± 57.730	101.500 ± 19.457	*t* = 1.101	0.271
BUN, M (Q_1_, Q_3_)	5.730 (4.628, 7.092)	5.700 (4.600, 6.998)	6.670 (5.107, 8.255)	Z = −2.331	0.020	5.720 (4.628, 7.090)	6.150 (5.163, 8.463)	Z = −0.902	0.367
Systolic pressure (mmHg), Mean ± SD	137.882 ± 18.315	137.663 ± 17.982	141.038 ± 22.764	*t* = −0.908	0.364	137.918 ± 18.384	136.125 ± 15.431	*t* = 0.274	0.784
Fracture type (Garden/Evans), *n* (%)				χ2 = 9.652	0.290			χ2 = 5.521	0.701
Garden	338 (84.500)	319 (94.379)	19 (5.621)			333 (98.521)	5 (1.479)		
Evans	62 (15.500)	55 (88.710)	7 (11.290)			59 (95.161)	3 (4.839)		
Hypertension, *n* (%)				χ2 = 1.310	0.252			χ2 = 0.722	0.396
No	257 (64.250)	243 (64.973)	14 (53.846)			253 (64.541)	4 (50.000)		
Yes	143 (35.750)	131 (35.027)	12 (46.154)			139 (35.459)	4 (50.000)		
Cancer, *n* (%)				χ2 = 0.423	0.515			χ2 = 6.685	0.010
No	394 (98.500)	368 (98.396)	26 (100.000)			387 (98.724)	7 (87.500)		
Yes	6 (1.500)	6 (1.604)	0 (0.000)			5 (1.276)	1 (12.500)		
Coronary heart disease, *n* (%)				χ2 = 0.004	0.948			χ2 = 0.209	0.647
No	325 (81.250)	304 (81.283)	21 (80.769)			319 (81.378)	6 (75.000)		
Yes	75 (18.750)	70 (18.717)	5 (19.231)			73 (18.622)	2 (25.000)		
Cerebrovascular disease, *n* (%)				χ2 = 0.865	0.352			χ2 = 0.004	0.950
No	347 (86.750)	326 (87.166)	21 (80.769)			340 (86.735)	7 (87.500)		
Yes	53 (13.250)	48 (12.834)	5 (19.231)			52 (13.265)	1 (12.500)		
Nervous system disease, *n* (%)				χ2 = 5.516	0.019			χ2 = 0.662	0.416
No	370 (92.500)	349 (93.316)	21 (80.769)			362 (92.347)	8 (100.000)		
Yes	30 (7.500)	25 (6.684)	5 (19.231)			30 (7.653)	0 (0.000)		
Respiratory system disease, *n* (%)				χ2 = 0.354	0.552			χ2 = 5.220	0.022
No	353 (88.250)	331 (88.503)	22 (84.615)			348 (88.776)	5 (62.500)		
Yes	47 (11.750)	43 (11.497)	4 (15.385)			44 (11.224)	3 (37.500)		
Digestive system disease, *n* (%)				χ2 = 0.207	0.649			χ2 = 16.954	< 0.001
No	390 (97.500)	365 (97.594)	25 (96.154)			384 (97.959)	6 (75.000)		
Yes	10 (2.500)	9 (2.406)	1 (3.846)			8 (2.041)	2 (25.000)		
Hepatic/Irenal insufficiency, *n* (%)				χ2 = 3.076	0.079			χ2 = 16.954	< 0.001
No	390 (97.500)	366 (97.861)	24 (92.308)			384 (97.959)	6 (75.000)		
Yes	10 (2.500)	8 (2.139)	2 (7.692)			8 (2.041)	2 (25.000)		
Diabetes, *n* (%)				χ2 = 0.037	0.847			χ2 = 1.642	0.200
No	333 (83.250)	311 (83.155)	22 (84.615)			325 (82.908)	8 (100.000)		
Yes	67 (16.750)	63 (16.845)	4 (15.385)			67 (17.092)	0 (0.000)		
Ectopic rhythm, *n* (%)				χ2 = 7.416	0.006			χ2 = 0.567	0.451
No	374 (93.500)	353 (94.385)	21 (80.769)			366 (93.367)	8 (100.000)		
Yes	26 (6.500)	21 (5.615)	5 (19.231)			26 (6.633)	0 (0.000)		
Tachyarrhythmian (%)				χ2 = 0.880	0.348			χ2 = 0.379	0.538
No	372 (93.000)	349 (93.316)	23 (88.462)			365 (93.112)	7 (87.500)		
Yes	28 (7.000)	25 (6.684)	3 (11.538)			27 (6.888)	1 (12.500)		
Conduction disorder, *n* (%)				χ2 = 2.184	0.702			χ2 = 0.869	0.929
No	350 (87.500)	325 (86.898)	25 (96.154)			343 (87.500)	7 (87.500)		
Atrial Premature Beats	27 (6.750)	26 (6.952)	1 (3.846)			26 (6.633)	1 (12.500)		
Supraventricular Premature Contractions	15 (3.750)	15 (4.011)	0 (0.000)			15 (3.827)	0 (0.000)		
Ventricular Premature Contractions	8 (2.000)	8 (2.139)	0 (0.000)			8 (2.041)	0 (0.000)		
Premature beat, *n* (%)				χ2 = 2.244	0.326			χ2 = 12.649	0.002
No	339 (84.750)	319 (85.294)	20 (76.923)			335 (85.459)	4 (50.000)		
Yes	61 (15.250)	55 (14.706)	6 (23.077)			53 (13.520)	3 (37.500)		
ECG band change, *n* (%)				χ2 = 3.228	0.072			χ2 = 1.762	0.184
No	329 (82.250)	311 (83.155)	18 (69.231)			321 (81.888)	8 (100.000)		
Yes	71 (17.750)	63 (16.845)	8 (30.769)			71 (18.112)	0 (0.000)		
Operation time (min), M (Q_1_, Q_3_)	80.000 (65.000, 105.000)	80.000 (65.000, 105.000)	60.000 (55.000, 85.000)	Z = 3.178	0.001	80.000 (65.000, 105.000)	135.000 (122.250, 153.750)	Z = −3.960	< 0.001
Bleeding volume (mL), *n* (%)				Z = 1.670	0.095			Z = −1.454	0.146
50	3 (0.750)	1 (0.267)	2 (7.692)			3 (0.765)	0 (0.000)		
100	28 (7.000)	27 (7.219)	1 (3.846)			28 (7.143)	0 (0.000)		
150	18 (4.500)	18 (4.813)	0 (0.000)			18 (4.592)	0 (0.000)		
200	82 (20.500)	72 (19.251)	10 (38.462)			80 (20.408)	2 (25.000)		
250	28 (7.000)	27 (7.219)	1 (3.846)			28 (7.143)	0 (0.000)		
300	74 (18.500)	70 (18.717)	4 (15.385)			72 (18.367)	2 (25.000)		
350	31 (7.750)	28 (7.487)	3 (11.538)			31 (7.908)	0 (0.000)		
400	60 (15.000)	57 (15.241)	3 (11.538)			60 (15.306)	0 (0.000)		
450	22 (5.500)	22 (5.882)	0 (0.000)			22 (5.612)	0 (0.000)		
500	21 (5.250)	21 (5.615)	0 (0.000)			19 (4.847)	2 (25.000)		
550	8 (2.000)	8 (2.139)	0 (0.000)			8 (2.041)	0 (0.000)		
600	6 (1.500)	6 (1.604)	0 (0.000)			6 (1.531)	0 (0.000)		
650	19 (4.750)	17 (4.545)	2 (7.692)			17 (4.337)	2 (25.000)		

Among the 1199 patients in the development cohort, the overall postoperative complication rate was 10.4% (125/1199) and the mortality rate was 1.8% (22/1199). Analysis of baseline characteristics revealed that mortality was significantly associated with advanced age (84.32 ± 7.45 vs. 78.67 ± 9.35 years, *p* = 0.001), impaired cardiac function (*p* = 0.015), fracture types (*p* = 0.038), cerebrovascular disease (*p* = 0.001), and respiratory system disease (*p* < 0.001). Postoperative complications were significantly correlated with digestive system disease (*p* = 0.022), hepatic/renal insufficiency (*p* = 0.037), and intraoperative bleeding volume (*p* = 0.027).

In the validation cohort (*n* = 400), the postoperative complication rate was 6.5% (26/400) and the mortality rate was 2.0% (8/400). Patients who developed complications were significantly older (median age: 83.00 [IQR: 80.25–86.50] vs. 77.00 [71.00–82.00] years, *p* < 0.001), had elevated blood urea nitrogen levels (6.67 [5.11–8.26] vs. 5.70 [4.60–7.00] mmol/L, *p* = 0.020), and showed higher prevalence of nervous system disease (*p* = 0.019) and ectopic rhythm (*p* = 0.006). Notably, operative time was significantly shorter in patients with complications (60.00 [55.00–85.00] vs. 80.00 [65.00–105.00] min, *p* = 0.001). Regarding mortality, significant associations were observed with digestive system disease (*p* < 0.001), hepatic/renal insufficiency (*p* < 0.001), premature beats (*p* = 0.002), respiratory system disease (*p* = 0.022), cancer (*p* = 0.010), and prolonged operative time (135.00 [122.25–153.75] vs. 80.00 [65.00–105.00] min, *p* < 0.001).

### 3.2 The Establishment of the New Prediction Model

The new DOORS‐GHF was: ln[R/(1 − R)] = −5.089 − 0.114 × OS+0.226 × PS, ln[Z/(1 − Z)] = −10.308 + 0.655 × OS + 0.201 × PS, R was the 30‐day postoperative complication, Z was the 30‐day postoperative mortality. PS represented the preoperative physiological scores, and OS represented the Operative severity score. The PS and OS item‐level scoring framework was adopted from DORSSSP v3.0 to preserve clinical familiarity, whereas the regression coefficients of the prediction equations were re‐estimated from the current dataset using binary logistic regression to optimize predictive accuracy for the geriatric hip fracture population. The predictive performance of DOORS‐GHF was assessed using ROC curves. For the prediction of the postoperative complication (Figure 2A), the AUC was 0.841, with a 95% confidence interval (CI) of 0.801–0.880. When it came to predicting postoperative mortality (Figure 2B), the AUC was 0.838, and the 95% CI was 0.753–0.923. Since both AUC values exceeded 0.7, this indicates that DOORS—GHF has good capability.

**FIGURE 2:**
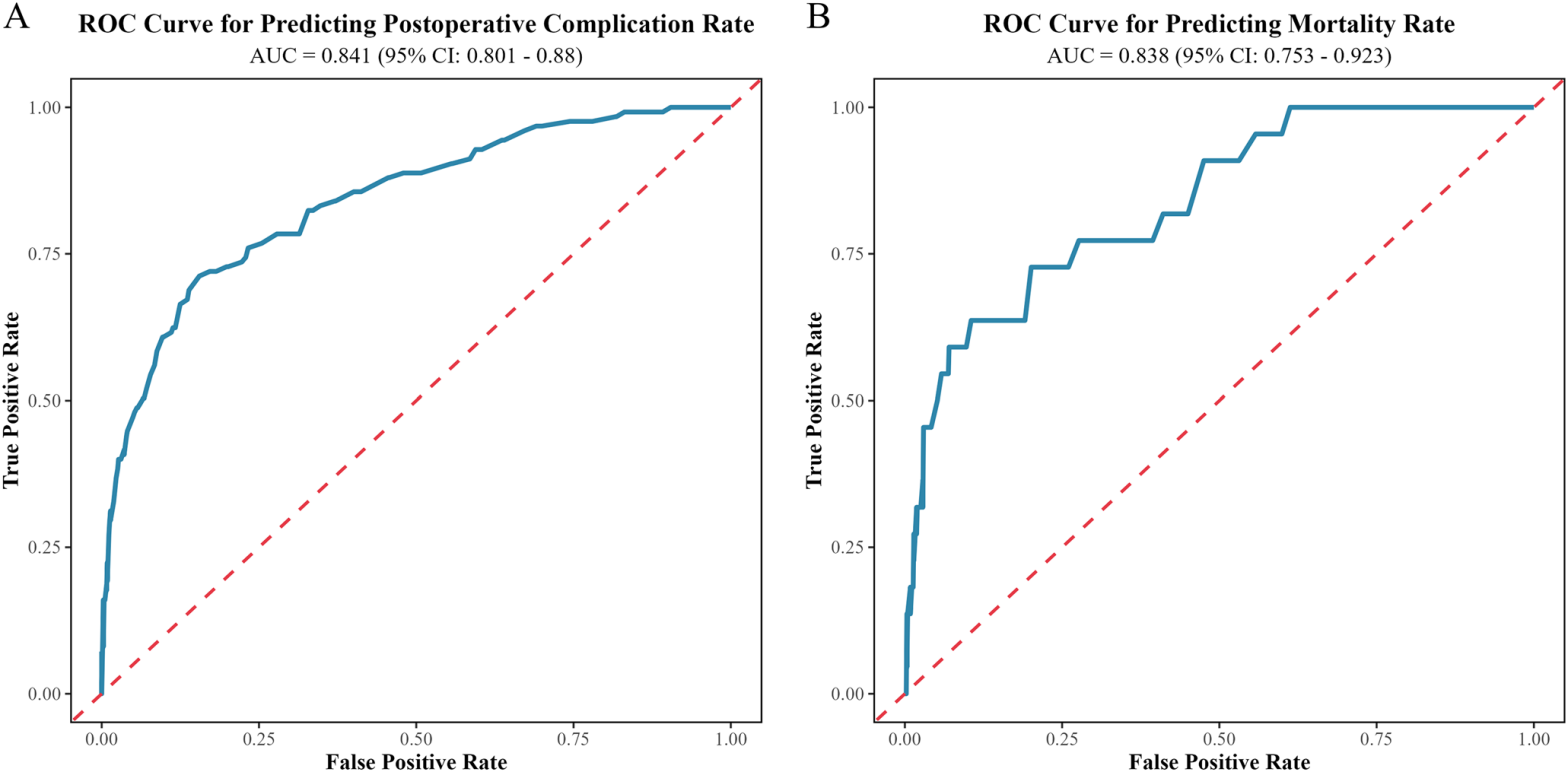
ROC curves of DOORS‐GHF for predicting 30‐day postoperative complications (A) and mortality (B) in the training cohort.

### 3.3 Comparison of DOORS‐GHF With DORSSSP v3.0 and P‐POSSUM in Predictive Performance

To evaluate the predictive performance of three scoring systems (DOORS‐GHF, DORSSSP v3.0, and P‐POSSUM) for postoperative complications and mortality, ROC curves and corresponding metrics (Table 6) (AUC, optimal cutoff value, sensitivity, and specificity) were analyzed. For predicting the postoperative complication rate (Figure 3A), DOORS‐GHF demonstrated the highest discriminative ability, with an AUC of 0.878 (95% CI: 0.821–0.935). This was significantly higher than the AUC of DORSSSP v3.0 (0.706, 95% CI: 0.612–0.799) and P‐POSSUM (0.776, 95% CI: 0.700–0.853). At the optimal cutoff value of 0.0988, DOORS‐GHF achieved a sensitivity of 0.8077 and specificity of 0.7701. For DORSSSP v3.0, the optimal cutoff was 0.0418 (sensitivity 0.6538, specificity 0.7139), while for P‐POSSUM, it was 0.0110 (sensitivity 0.9615, specificity 0.4893). In predicting postoperative mortality (Figure 3B), DOORS‐GHF again showed the strongest discriminative performance (AUC = 0.968, 95% CI: 0.912–1.000), outperforming DORSSSP v3.0 (AUC = 0.857, 95% CI: 0.676–1.000) and P‐POSSUM (AUC = 0.812, 95% CI: 0.648–0.976). However, the wide confidence interval—reflecting the low mortality event rate (8/400, 2.0%)—warrants cautious interpretation. The optimal cutoff for DOORS‐GHF was 0.0672, yielding a sensitivity of 0.8750 and perfect specificity (0.9847). DORSSSP v3.0 had an optimal cutoff of 0.2502 (sensitivity 0.8750, specificity 0.8163), and P‐POSSUM had a cutoff of 0.0309 (sensitivity 0.6250, specificity 0.9617). These results indicate that DOORS‐GHF exhibited superior predictive performance for both postoperative complication rate and mortality. For the complication rate, DOORS‐GHF achieved moderate to good discrimination (AUC = 0.878), while for mortality, it showed excellent discrimination (AUC > 0.9), although this estimate should be interpreted cautiously given the limited number of events (8/400). In contrast, DORSSSP v3.0 and P‐POSSUM had lower discriminative abilities in both scenarios, with AUCs ranging from 0.706 to 0.857. The violin plots structure highlights the differences in predicted probability distributions (Figure 4) and the statistical characteristics of actual outcomes (Table 7) across the three models.

**TABLE 6:** Predictive performance of the three scoring systems in the external validation cohort.

Scoring system	Postoperative complication rate					Postoperative mortality				
	Optimal cutoff value	Sensitivity	Specificity	AUC	95% CI	Optimal cutoff value	Sensitivity	Specificity	AUC	95% CI
DOORS‐GHF	0.0988	0.8077	0.7701	0.8780	0.821–0.935	0.0672	0.8750	0.9847	0.9680	0.912–1.000
DORSSSPv3.0	0.0418	0.6538	0.7139	0.7060	0.612–0.799	0.2502	0.8750	0.8163	0.8570	0.676–1.000
P‐POSSUM	0.0110	0.9615	0.4893	0.7760	0.700–0.853	0.0309	0.6250	0.9617	0.8120	0.648–0.976

**FIGURE 3:**
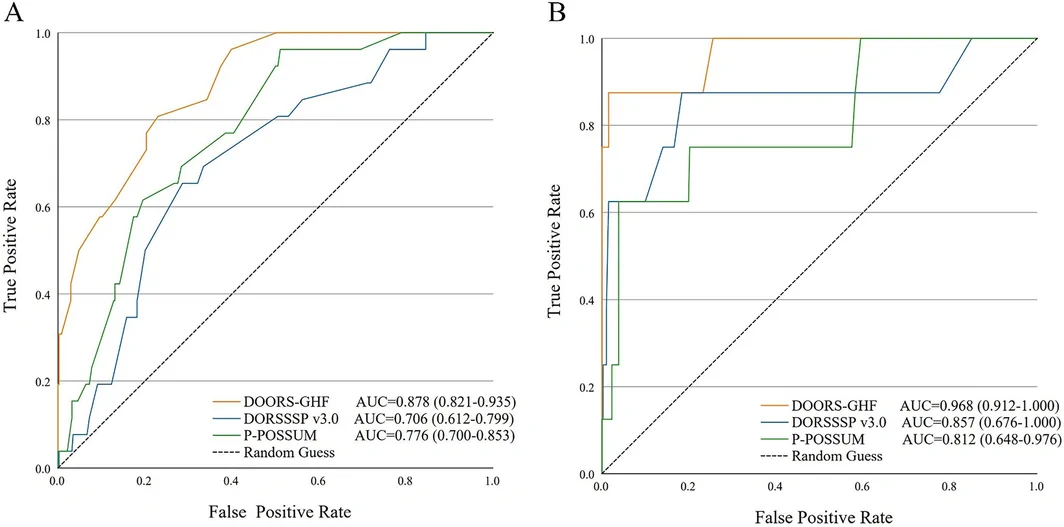
ROC curves of DOORS‐GHF, DORSSSP v3.0, and P‐POSSUM for predicting 30‐day postoperative complications (A) and mortality (B) in the validation cohort.

**FIGURE 4:**
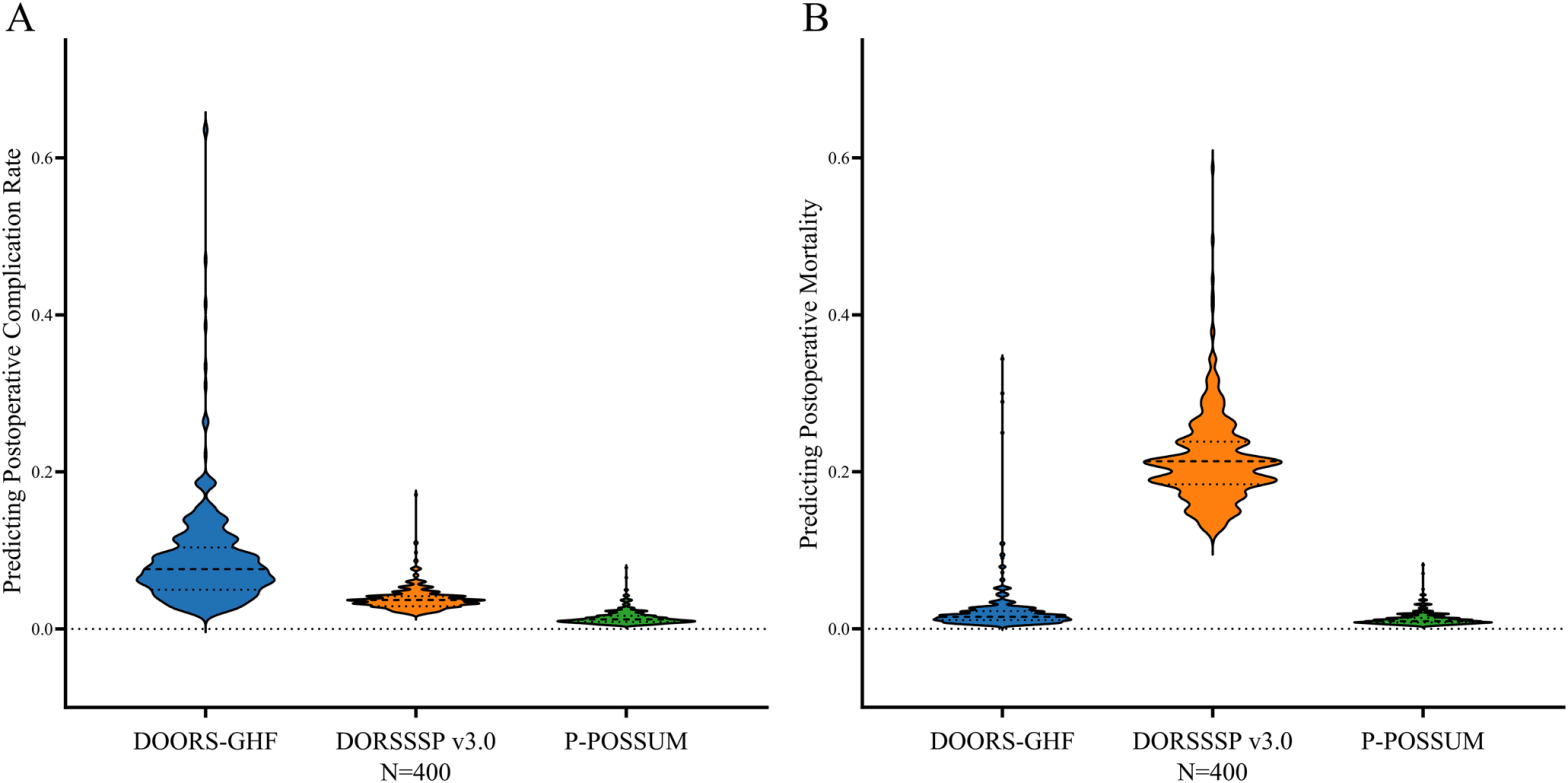
Violin plots of DOORS‐GHF, DORSSSP v3.0, and P‐POSSUM for 30‐day postoperative complications (A) and mortality (B) in the validation cohort.

**TABLE 7:** Predictive probability distribution of the three scoring systems in the external validation cohort.

Statistical metric	Postoperative complication rate			Postoperative mortality		
	DOORS‐GHF	DORSSSP v3.0	P‐POSSUM	DOORS‐GHF	DORSSSP v3.0	P‐POSSUM
Minimum	0.0186	0.0174	0.0043	0.0034	0.1173	0.0036
Maximum	0.6358	0.1709	0.0780	0.3439	0.5870	0.0813
Range	0.6172	0.1535	0.0737	0.3405	0.4697	0.0777
25th percentile	0.0499	0.0288	0.0089	0.0113	0.1840	0.0082
Median	0.0762	0.0369	0.0123	0.0153	0.2135	0.0099
75th percentile	0.1039	0.0419	0.0168	0.0226	0.2384	0.0138
Mean	0.0881	0.0381	0.0143	0.0222	0.2136	0.0125

This analysis highlighted DOORS‐GHF as the most accurate tool for predicting postoperative outcomes in this dataset, outperforming existing models (DORSSSP v3.0 and P‐POSSUM) in terms of overall predictive power (AUC) and balanced sensitivity/specificity at optimal cutoffs.

DOORS‐GHF demonstrated superior predictive accuracy and calibration compared with DORSSSP v3.0 and P‐POSSUM across both postoperative outcomes (Table 8). For complication prediction, DOORS‐GHF achieved good calibration (Hosmer–Lemeshow χ^2^ = 11.887, df = 8, *p* = 0.156) and the lowest Brier score (0.0489), outperforming DORSSSP v3.0 (HL χ^2^ = 13.457, df = 7, *p* = 0.062; Brier = 0.0605) and P‐POSSUM, which exhibited significant calibration failure (HL χ^2^ = 16.560, df = 7, *p* = 0.020; Brier = 0.0625). For mortality prediction, DOORS‐GHF maintained good calibration (HL χ^2^ = 6.259, df = 7, *p* = 0.510) with the lowest Brier score (0.0140), compared with DORSSSP v3.0 (HL χ^2^ = 9.027, df = 7, *p* = 0.251; Brier = 0.0548) and P‐POSSUM (HL χ^2^ = 8.724, df = 7, *p* = 0.273; Brier = 0.0190). These findings confirm the consistent performance of DOORS‐GHF, with the lowest Brier scores and adequate calibration (non‐significant HL tests) across both complication and mortality predictions.

**TABLE 8:** Calibration and goodness‐of‐fit of the three scoring systems in the external validation cohort.

Scoring system	Postoperative complication rate				Postoperative mortality			
	Hosmer‐Lemeshow (HL) test			The Brier score	Hosmer‐Lemeshow (HL) test			The Brier score
	The chi‐square value	The degrees of freedom	*p*‐value		The chi‐square value	The degrees of freedom	*p*‐value	
DOORS‐GHF	11.887	8	0.156	0.0489	6.259	7	0.510	0.0140
DORSSSP v3.0	13.457	7	0.062	0.0605	9.027	7	0.251	0.0548
P‐POSSUM	16.560	7	0.020	0.0625	8.724	7	0.273	0.0190

### 3.4 Comparison of DOORS‐GHF With DORSSSP v3.0 and P‐POSSUM in Clinical Value

To assess the clinical utility of the DOORS‐GHF, DORSSSP v3.0, and P‐POSSUM models in predicting postoperative outcomes, DCA was performed. DCA evaluates the net benefit of a model across various threshold probabilities, where net benefit quantifies the clinical value of using the model to guide decisions (such as intervening for patients with predicted risk≥threshold) compared to the treat‐all or the treat‐none strategies. A higher net benefit indicates greater clinical utility. Bootstrap resampling (1000 iterations) was performed to estimate 95% confidence bands for the DCA curves, confirming the stability of net benefit estimates across clinically relevant threshold ranges.

DOORS‐GHF (Figure 5A): The model demonstrated superior net benefit compared to the treat‐none strategy (y = 0) across threshold probabilities of 0–0.65, with a peak net benefit of approximately 0.075. It consistently outperformed the treat‐all strategy within this range. However, net benefit turned negative at higher thresholds (> 0.65), indicating excessive intervention risks.

**FIGURE 5:**
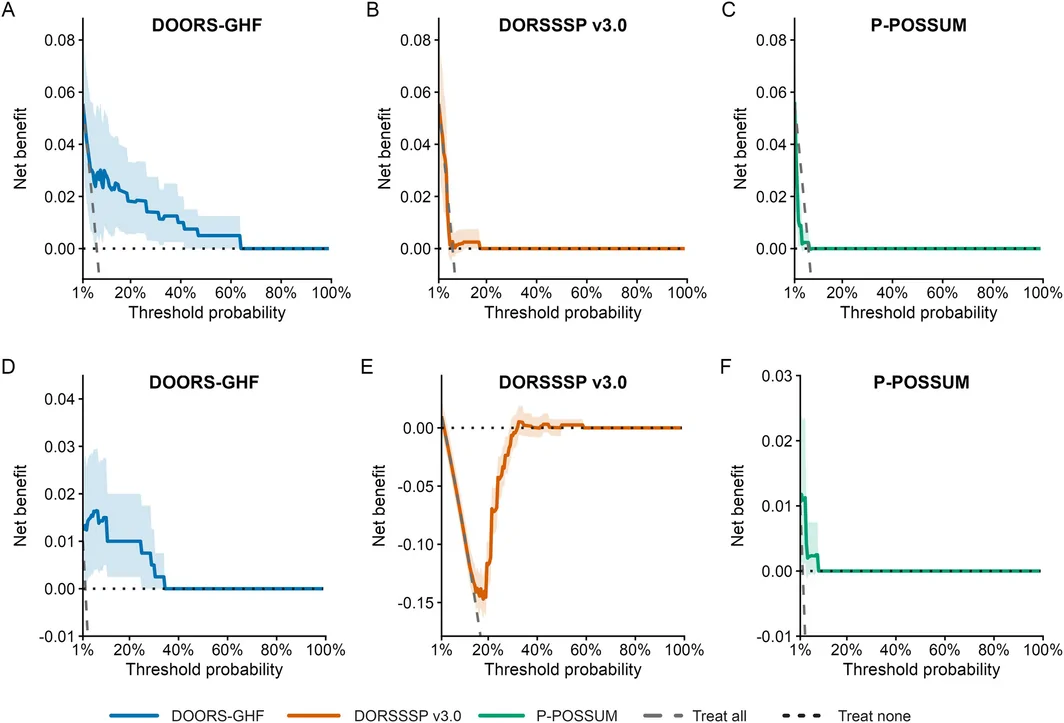
Decision curve analysis (DCA) of DOORS‐GHF, DORSSSP v3.0 and P‐POSSUM for 30‐day postoperative complications (A‐C) and mortality (D‐F) in the validation cohort.

DORSSSP v3.0 (Figure 5B): The model showed negligible net benefit across virtually all threshold probabilities, with the curve remaining close to the treat‐none strategy (y = 0). The peak net benefit was approximately 0.01 at threshold 0, but the model failed to provide meaningful incremental benefit above either the treat none or treat all strategies, indicating minimal clinical utility for complication prediction.

P‐POSSUM (Figure 5C): The model exhibited an initial net benefit of approximately 0.08 at threshold 0, but this declined rapidly and became negative beyond a threshold of 0.05. Thus, clinical utility was limited to a very narrow low‐threshold range (0–0.05), with no added value at intermediate or higher thresholds.

DOORS‐GHF (Figure 5D): The model maintained positive net benefit above both the treat‐all and the treat‐none strategies across threshold probabilities of 0–0.40. It demonstrated robust clinical utility for thresholds below 0.6, with a peak net benefit of approximately 0.018, though losing incremental benefit at higher thresholds (> 0.40).

DORSSSP v3.0 (Figure 5E): DCA revealed clinical utility only within the narrow intermediate threshold range of 0.32–0.57, with net benefit approaching 0.01. The model exhibited substantial overtreatment risks (negative net benefit) at low thresholds (< 0.30), and no net benefit at high thresholds (> 0.57), limiting practical applicability to this narrow window.

P‐POSSUM (Figure 5F): Positive net benefit was observed only within the low‐threshold range of 0.01–0.15, with a maximum benefit of approximately 0.015. Net benefit rapidly declined to the treat none level beyond a threshold of 0.15, indicating minimal clinical utility in this narrow interval.

DCA results demonstrated that DOORS‐GHF exhibited the broadest and most stable clinical utility for predicting postoperative complications (0–0.65) and mortality (0–0.40). In contrast, both DORSSSP v3.0 and P‐POSSUM models showed restricted application ranges with limited benefit magnitude—DORSSSP v3.0 demonstrated negligible utility for complications and only marginal utility in a narrow mortality threshold range, while P‐POSSUM exhibited a high initial net benefit for complications but only within a very narrow low‐threshold window, and minimal utility for mortality. These findings establish DOORS‐GHF as the most valuable clinical decision‐making tool for postoperative risk assessment in geriatric hip fracture patients, although the mortality‐related conclusions warrant cautious interpretation given the limited number of events.

## 4 Discussion

### 4.1 Principal Findings

In this study, a novel risk scoring system (DOORS‐GHF) was successfully developed and validated specifically for Chinese geriatric patients with hip fractures. The system was developed using a multicenter development cohort of 1199 patients and externally validated in an independent temporally‐separated cohort of 400 patients. Validation results demonstrated that DOORS‐GHF exhibits superior discrimination, calibration, predictive accuracy, and clinical net benefit compared with the widely used DORSSSP v3.0 and P‐POSSUM scoring systems in predicting 30‐day postoperative complications and mortality in geriatric hip fracture patients, thereby filling a critical gap in dedicated risk scoring tools for geriatric hip fractures in China.

### 4.2 Comparison With Established Scoring Systems

Our findings corroborated the limitations of established systems in the geriatric hip fracture population. Johns et al. reported that 50% of patients who died after hip fracture surgery were classified as very low risk (0%–10%) by the P‐POSSUM system, indicating that this system failed to accurately identify high‐risk patients [19]. A meta‐analysis by Yang et al. also demonstrated that P‐POSSUM significantly overestimated mortality in geriatric hip fracture patients, with an observed/expected mortality ratio of 0.61 [40]. In our external validation, the Brier score for mortality prediction by P‐POSSUM was 0.0190, markedly higher than that of DOORS‐GHF (0.0140), reflecting the insufficient predictive accuracy of the established system. For the widely used Chinese DORSSSP v3.0 system, although it was better adapted to the Chinese population than foreign systems, it was developed for the entire geriatric orthopedic surgical population and thus lacked specificity for hip fracture patients. In our study, its areas under the ROC curves for predicting complications and mortality in the external validation cohort were 0.706 and 0.857, respectively, both substantially lower than those of DOORS‐GHF (0.878 and 0.968). DCA showed that DORSSSP v3.0 had an extremely narrow—or even near‐zero—range of threshold probabilities with positive net benefit compared to DOORS‐GHF, particularly for complication prediction (Figure 5B). In contrast, DOORS‐GHF achieved an AUC of 0.878 for complication prediction and 0.968 for mortality prediction in the 400‐patient external validation cohort. Among the three systems, DOORS‐GHF simultaneously showed the best calibration (Hosmer‐Lemeshow *p* = 0.156 and 0.510), lowest prediction error (Brier scores of 0.0489 and 0.0140), and widest clinical applicability (DCA net benefit across threshold probabilities of 0–0.65 for complications and 0–0.40 for mortality), fully demonstrating its predictive superiority in the geriatric hip fracture population.

It should be emphasized that the preoperative physiological score (PS) plus operative severity score (OS) dual‐score framework employed by DOORS‐GHF represents a classic, universally validated paradigm for surgical risk assessment, rather than an exclusive structural feature of any specific system. We adopted this mature architecture to ensure clinical usability and horizontal comparability with mainstream tools; the adoption of a shared framework does not negate the population specificity of our model. The specificity of DOORS‐GHF is fundamentally grounded in three dimensions. First, systematic reconstruction of the indicator system for geriatric hip fracture characteristics: we removed indicators weakly associated with this population from P‐POSSUM (e.g., Glasgow Coma Scale, serum sodium/potassium), and incorporated population‐specific core predictors including the age‐adjusted Charlson Comorbidity Index (aCCI), fracture type (femoral neck vs. intertrochanteric), diabetes grading, and cardiac function classification, all rigorously defined with explicit criteria and references (Tables [Link], [Link]). The operative severity grading was also recalibrated based on common hip fracture surgical procedures. Second, independent weight estimation: all model coefficients were derived de novo from 1199 Chinese geriatric hip fracture patients using binary logistic regression, rather than being re‐weighted from existing models. Notably, the OS regression coefficient for mortality prediction (0.655) was substantially higher than that in the DORSSSP v3.0 system (0.1853), accurately capturing the clinical reality that surgical trauma exerts a disproportionately greater impact on frail elderly patients. Third, the significantly superior predictive performance, confirmed in both the temporally independent external validation involving 400 patients (AUC 0.878/0.968) and the imputation sensitivity analysis involving 630 patients (AUC 0.952/0.948), provides direct empirical evidence that population‐specific indicator optimization and weight recalibration produce clinically meaningful improvements beyond those achievable by simple recalibration of generic systems.

Previous studies have confirmed inherent interethnic differences in baseline physiology, chronic disease spectrum, drug metabolic capacity, and physiological reserve, all of which directly affect patients' tolerance to surgical stress and postoperative clinical outcomes [41]. For example, the cardiovascular disease spectrum, diabetes prevalence, and baseline BMI in the Chinese geriatric population differ markedly from those in Western geriatric populations [42]. However, the P‐POSSUM system was developed using a North American and European general surgical population, with parameter thresholds and weight assignments based on the disease characteristics of this population, leading to an inherent mismatch with the physiological characteristics of the Chinese population [43]. This was the core mechanism underlying the widespread risk overestimation of this system in Chinese populations [44]. Such risk overestimation may lead to excessively conservative clinical decision‐making, potentially depriving patients who could tolerate surgery of the opportunity for early surgical intervention—a strategy proven to reduce long‐term mortality in this population. Meanwhile, the rapid development of China's healthcare capacity and changes in population structure have created an imperative for the iterative updating of risk scoring systems [45]. The DORSSSP system was previously developed using modeling data from Chinese orthopedic patients collected over a decade ago [46]. Over the past 10 years, the life expectancy of the Chinese population has increased significantly, the proportion of very elderly hip fracture patients aged ≥ 75 years has risen sharply, and the spectrum of underlying diseases, perioperative anesthesia management protocols, and enhanced recovery after surgery strategies have undergone fundamental changes [47]. The parameter weights of the original model were no longer adequate for the clinical characteristics of contemporary patients, leading to deviations in risk prediction. The DOORS‐GHF system developed in this study was constructed using recent multicenter clinical data from Chinese geriatric hip fracture patients, addressing the issue of population adaptability from the source of model development, which underpins its significantly superior predictive performance over existing systems [48].

### 4.3 The Role of Comorbidity Burden (aCCI)

The cumulative burden of comorbidities is a critical determinant of perioperative adverse event risk in geriatric hip fracture patients, and its impact on prognosis often exceeds that of surgical trauma itself [49]. This was the core consideration for incorporating the aCCI into the model. Geriatric hip fracture patients are predominantly very elderly and often present with multiple chronic conditions, including cardiovascular disease, chronic respiratory disease, diabetes mellitus, and chronic renal insufficiency [50]. These conditions do not exist in isolation; rather, they act synergistically to reduce patients' physiological reserve and stress tolerance [51]. Multiple chronic diseases place patients in a state of chronic low‐grade inflammation, and surgical trauma further disrupts physiological homeostasis, triggering multiorgan decompensation and ultimately leading to postoperative complications or even death. Previous studies have also confirmed that geriatric hip fracture patients with three or more comorbidities have a 2.5‐fold higher 30‐day postoperative mortality rate than those without comorbidities [52]. However, existing scoring systems have significant shortcomings in the systematic assessment of comorbidity burden: the P‐POSSUM system only incorporates organ‐specific functional parameters such as cardiac and renal indicators, failing to provide a comprehensive evaluation of the patient's overall comorbid status, while the DORSSSP system includes some disease‐related indicators; it inadequately accounts for the cumulative burden of comorbidities, making it difficult to accurately quantify the impact of multimorbidity on prognosis [53]. The aCCI incorporated in this study, which was optimized by adding age weighting to the classic CCI, enables quantitative assessment of the cumulative impact of 19 common chronic diseases on patient prognosis, while accounting for the decline in physiological reserve associated with advancing age [54]. It has been validated as a reliable tool for predicting prognosis in geriatric surgical patients by numerous studies [55]. The incorporation of the Age‐Adjusted Charlson Comorbidity Index (aCCI) markedly improved the ability of the DOORS‐GHF system to identify high‐risk patients with multiple comorbidities, addressing the gap in comorbidity risk assessment in existing systems [56].

### 4.4 Optimization of the Operative Severity Score for Hip Fracture Surgery

Hip fracture surgery differs fundamentally from general surgery and conventional elective orthopedic surgery [57]. This difference is the core rationale for optimizing the parameters and weights of the OS (the operative severity score). In terms of commonalities, all surgeries trigger a systemic stress response in response to surgical trauma, and the extent of surgical trauma is positively correlated with the risk of postoperative adverse events [58]. However, geriatric hip fracture surgery has distinct clinical characteristics: first, patients are predominantly very elderly and frail, with markedly reduced physiological reserve, and have far lower tolerance to surgical trauma than the general surgical population; second, most of these surgeries are time‐sensitive, with a short preoperative preparation window, making it difficult to optimize the patient's baseline status through long‐term prehabilitation, resulting in higher surgery‐related risk; finally, surgical approaches are relatively fixed, mainly consisting of arthroplasty and closed reduction with internal fixation, with an extremely low incidence of general surgery‐related risk factors such as surgical contamination and multiple procedures [59]. The operative severity parameter settings of existing scoring systems do not account for the above characteristics of hip fracture surgery [60]. The P‐POSSUM system, derived from general surgery, incorporates parameters with low relevance to hip fracture surgery, such as operative severity, peritoneal contamination, and multiple procedures, which not only increase the complexity of clinical assessment but also predispose to systematic bias in risk evaluation [61]. The DORSSSP v3.0 system was designed for all orthopedic surgeries, with weight assignments for operative parameters not calibrated for the specific features of hip fractures, making it difficult to accurately reflect the true impact of surgical trauma on this patient population [62]. Previous studies have confirmed that operative duration and intraoperative blood loss are the most direct reflections of surgical trauma in hip fracture surgery, and are significantly positively correlated with postoperative blood transfusion rate, infection risk, and complication rates [28]. In this study, we simplified the operative severity parameters of DOORS‐GHF to three core indicators: surgical approach, operative duration, and intraoperative blood loss—all objective indicators readily available in clinical practice. Meanwhile, we recalibrated the weights of the OS score via regression analysis [63]. The results showed that the regression coefficient of OS in the mortality prediction equation was 0.655, significantly higher than that in previous models, indicating that surgery‐related factors exert a greater impact on mortality in geriatric hip fracture patients [64]. The weight optimization in this study aligns the model more closely with the clinical rules of hip fracture surgery, fundamentally resolving the issues of suboptimal operative parameter settings and mismatched weights in the original systems.

### 4.5 Choice of the Modeling Approach

In this study, the prediction equation was constructed using a binary logistic regression model, the most widely used and standardized method for developing clinical risk prediction models. Compared with complex algorithms such as machine learning and deep learning, binary logistic regression has distinct clinical advantages: first, the model has strong interpretability, as the regression coefficient of each variable directly reflects its relative contribution to the outcome, enabling clinicians to clearly understand the contribution of each indicator to risk; second, the formula is concise with a low barrier to clinical application—risk assessment can be completed rapidly by calculating only the PS and OS, without relying on complex computational tools, making it more suitable for promotion in hospitals at all levels; finally, the classic POSSUM‐series scoring systems and the DORSSSP system were all constructed using logistic regression, and our adoption of the same methodological framework ensures comparability with existing systems and facilitates clinician understanding and acceptance.

### 4.6 Sensitivity Analysis

To further assess the robustness of our findings and rule out potential selection bias introduced by the exclusion of patients with incomplete clinical data, we performed a comprehensive sensitivity analysis on the 630 patients (28.3% of the eligible population) who were excluded from the development cohort due to missing clinical variables. All these patients had a variable‐level missing rate below 30%, meeting the recommended threshold for multiple imputation. We applied random forest‐based multiple imputation—using the methodology described in our previously published domain‐adaptation study (PMID: 40990498)—to generate a fully imputed dataset for all 630 patients. A baseline comparison between the complete cohort (*N* = 1599) and the imputation cohort (*N* = 630) across 24 demographic, clinical, laboratory, and surgical variables demonstrated that the two cohorts were broadly comparable (Table S1). Statistically significant differences in cardiac function classification and several comorbidity rates were consistent with the expected clinical pattern in retrospective registries, wherein patients with more severe conditions receive more comprehensive examinations and thus have more complete data records. Crucially, full validation of DOORS‐GHF on the imputation cohort confirmed its stable and excellent predictive performance (Figures [Link], [Link], [Link]): the AUC was 0.952 (95% CI: 0.930–0.974) for 30‐day complications and 0.948 (95% CI: 0.917–0.978) for 30‐day mortality (Table S2), both highly consistent with the external validation results in the complete cohort (0.878 and 0.968, respectively). Calibration performance and clinical net benefit evaluated by decision curve analysis were likewise maintained, with no evidence of performance degradation attributable to data missingness. This sensitivity analysis provides robust evidence that the predictive performance of DOORS‐GHF is not driven by selection bias arising from the exclusion of incomplete cases, and that the conclusions regarding its superior performance are reliable and stable across both complete‐case and imputed‐data analytic frameworks.

### 4.7 Strengths and Limitations

This study has several notable strengths. First, it leverages one of the largest contemporary datasets (*N* = 1599) specifically assembled for geriatric hip fracture risk prediction in China. Second, the scoring system was externally validated in a temporally independent cohort of 400 patients, with an additional sensitivity analysis on 630 imputed cases confirming result robustness against selection bias. Third, the integration of the age‐adjusted Charlson Comorbidity Index (aCCI)—a parameter absent from the original POSSUM framework—directly improved predictive accuracy by capturing the cumulative burden of comorbidities. Fourth, the PS + OS dual‐score structure preserves the classic POSSUM paradigm while enabling bedside computation without reliance on complex software, facilitating adoption in hospitals at all tiers. Fifth, temporal splitting of development and validation cohorts reflects real‐world clinical deployment and reduces over‐optimism in internal performance estimates.

There are several limitations in this study. First, it is a retrospective, multicenter study. Although strict inclusion and exclusion criteria and data extraction quality control specifications were established, the inherent selection bias and information bias of retrospective studies cannot be completely avoided. We are further validating the predictive performance of this system through prospective studies. Second, the modeling and validation data of this study were all derived from Chinese tertiary (Grade A) hospitals, and the applicability of the model in primary‐level hospitals requires further validation, as perioperative management models and baseline health status of patients in primary hospitals differ from those in tertiary hospitals. In addition, this study only evaluated the predictive performance of the model for 30‐day postoperative complications and mortality and did not explore its predictive value for long‐term outcomes such as 1‐year mortality and postoperative functional recovery. Finally, this study did not incorporate emerging prognostic indicators such as frailty scores and preoperative inflammatory biomarkers, which can be included in future studies to further optimize the predictive performance of the model. Furthermore, accurate quantification of intraoperative blood loss remains a common challenge in retrospective clinical studies. The subgroup mean estimation method adopted in this study may have certain individual deviations, which could exert a potential impact on the accuracy of the scoring system. In subsequent prospective studies, we will adopt more standardized and accurate blood loss calculation methods (such as measurement via graduated suction canisters, the gravimetric method for surgical gauze, and the hemoglobin difference‐based calculation method) to further improve data quality and model reliability.

## 5 Conclusion

In summary, the DOORS‐GHF system developed in this study is specifically designed for Chinese geriatric patients with hip fractures. Targeting the core limitations of existing systems, we completed model refinement across three dimensions: population adaptability through contemporary multicenter data, comprehensive comorbidity risk assessment via aCCI integration, and optimization of operative severity weights specifically calibrated for hip fracture procedures. The system's robust predictive performance has been demonstrated across multiple independent cohorts—a 400‐patient temporally independent external validation and a 630‐patient multiple imputation sensitivity analysis—consistently exhibiting superior discrimination, calibration, and clinical net benefit relative to the DORSSSP v3.0 and P‐POSSUM systems. These findings support DOORS‐GHF as a reliable, clinically practical risk stratification tool for perioperative decision‐making in geriatric hip fracture patients, and provide a foundation for its prospective validation and eventual clinical implementation.

## Author Contributions

**Zhiwei Gong:** conceptualization, methodology, software, data curation, writing – original draft, investigation, writing – review and editing, validation, formal analysis. **Yu Wang:** conceptualization, methodology, software, data curation, investigation, writing – original draft, writing – review and editing, validation, formal analysis. **Weiwen Zhu:** conceptualization, methodology, software, data curation, investigation, writing – original draft, validation, formal analysis. **Jing Zhong:** conceptualization, methodology, software, data curation, investigation. **Xiangdong Liu:** conceptualization, methodology, investigation, data curation. **Runze Jin:** conceptualization, investigation, methodology, data curation. **Zhaoyuan Zhang:** conceptualization, investigation, methodology, data curation. **Kesi Liu:** conceptualization, investigation, methodology, data curation. **Ziming Wang:** conceptualization, investigation, methodology, software, data curation, writing – review and editing. **Xingchen Lu:** conceptualization, methodology, software, data curation, investigation, writing – review and editing. **Yan Xiong:** conceptualization, methodology, software, data curation, investigation, funding acquisition, resources, writing – review and editing.

## Funding

The present study was supported by the Medical Research Project jointly funded by the Science‐Health Joint Medical Scientific Research Project of Chongqing, China (2022ZDXM011) and (2026ZDXM020).

## Ethics Statement

Ethical approval for this study (Ethical Approval No. 331) was provided by the Medical Research Ethics Review Committee of the PLA Army Medical Center, Chongqing, China on 20 November 2024.

## Conflicts of Interest

The authors declare no conflicts of interest.

## Supporting information


**Figure S1:** ROC curves of DOORS‐GHF, DORSSSP v3.0 and P‐POSSUM for predicting 30‐day postoperative complications (A) and mortality (B) in the supplementary validation cohort.

**Figure S2:** Violin plots of DOORS‐GHF, DORSSSP v3.0 and P‐POSSUM for 30‐day postoperative complications (A) and mortality (B) in the supplementary validation cohort.

**Figure S3:** Decision curve analysis (DCA) of DOORS‐GHF, DORSSSP v3.0 and P‐POSSUM for 30‐day postoperative complications (A–C) and mortality (D–F) in the supplementary validation cohort.

**Table S1:** Baseline characteristics comparison: complete cohort (*N* = 1599) vs. imputation cohort (*N* = 630).

**Table S2:** AUC comparison between complete validation cohort and imputation cohort.

**Table S3:** Diabetes classification.

**Table S4:** Cardiac function classification.

**Table S5:** Pulmonary function classification.

**Table S6:** Surgical grading.

**Table S7:** Age‐adjusted charlson comorbidity index (aCCI).

**Table S8:** Estimated intraoperative blood loss by surgical procedure and age group.

## Data Availability

The data that support the findings of this study are available on request from the corresponding author. The data are not publicly available due to privacy or ethical restrictions.
