# Supplementary material for: Development and Validation of Daping Orthopedics Operative Risk Scoring System for Geriatric Hip Fracture (DOORS‐GHF) for Predicting Postoperative Outcomes in Geriatric Hip Fracture Patients: A Multicenter Retrospective Comparative Study
Source: Orthop Surg. 2026 Sep 27;18(11):2356–79. doi: 10.1111/os.70430 (PMC13616831; doi:10.1111/os.70430)
Supplement: Supplementary file 2 [file OS-18-2356-s004.docx]

**SUPPLEMENTARY TABLE 2 | AUC comparison between complete validation cohort and imputation cohort.**

| **Outcome / Cohort** | **DOORS-GHF AUC (95% CI)** | **DORSSSP v3.0 AUC (95% CI)** | **P-POSSUM AUC (95% CI)** |
| --- | --- | --- | --- |
| **30-day Postoperative Complications** |  |  |  |
| Complete validation cohort (N=400) | **0.878** (0.821-0.935) | 0.706 (0.612-0.799) | 0.776 (0.700-0.853) |
| Imputation cohort (N=630) | **0.952** (0.930-0.974) | 0.693 (0.628-0.759) | 0.764 (0.699-0.828) |
| **30-day Postoperative Mortality** |  |  |  |
| Complete validation cohort (N=400) | **0.968** (0.912-1.000) | 0.857 (0.676-1.000) | 0.812 (0.648-0.976) |
| Imputation cohort (N=630) | **0.948** (0.917-0.978) | 0.818 (0.693-0.944) | 0.842 (0.712-0.972) |

Note: Values are presented as decimal.

Abbreviations: AUC, area under the receiver operating characteristic curve; CI, confidence interval.
