# Supplementary material for: Development and Validation of Daping Orthopedics Operative Risk Scoring System for Geriatric Hip Fracture (DOORS‐GHF) for Predicting Postoperative Outcomes in Geriatric Hip Fracture Patients: A Multicenter Retrospective Comparative Study
Source: Orthop Surg. 2026 Sep 27;18(11):2356–79. doi: 10.1111/os.70430 (PMC13616831; doi:10.1111/os.70430)
Supplement: Supplementary file 3 [file OS-18-2356-s005.docx]

**SUPPLEMENTARY TABLE 7 | Age-Adjusted Charlson Comorbidity Index (aCCI).**

| **Point A (Comorbidity Weight)** | **Charlson Comorbidity Index-Comorbidities and Weights** |
| --- | --- |
| 1 | Myocardial infarct; Congestive heart failure; Peripheral vascular disease; Cerebrovascular disease; Dementia; Chronic pulmonary disease; Connective tissue disease; Ulcer disease; Mild liver disease; Diabetes |
| 2 | Hemiplegia; Moderate or severe renal disease; Diabetes with end organ damage; Any tumor; Leukemia; Lymphoma |
| 3 | Moderate or severe liver disease |
| 6 | Metastatic solid tumor; AIDS |
| **Point B (Age Adjustment)** | **Age Range** |
| 0 | ＜50 years |
| 1 | 50-59 years |
| 2 | 60-69 years |
| 3 | 70-79 years |
| **Total aCCI score = Point A (Comorbidity Weight) + Point B (Age Adjustment)** | |
