# Supplementary material for: Development and Validation of Daping Orthopedics Operative Risk Scoring System for Geriatric Hip Fracture (DOORS‐GHF) for Predicting Postoperative Outcomes in Geriatric Hip Fracture Patients: A Multicenter Retrospective Comparative Study
Source: Orthop Surg. 2026 Sep 27;18(11):2356–79. doi: 10.1111/os.70430 (PMC13616831; doi:10.1111/os.70430)
Supplement: Supplementary file 5 [file OS-18-2356-s008.docx]

**SUPPLEMENTARY TABLE 5 | Pulmonary Function Classification.**

| **Pulmonary Function Classification** | **Specific Contents of Pulmonary Function Classification** |
| --- | --- |
| A (Class I) | Except during strenuous exercise, no significant dyspnea; or breathlessness when walking fast on the level or walking up a slight hill. |
| B (Class II) | Due to dyspnea, walks slower than people of the same age on the level or has to stop for breath when walking at own pace on the level. |
| C (Class III) | Stops for breath after walking about 100 meters or after a few minutes on the level. |
| D (Class IV) | Too breathless to leave the house, or breathless when dressing or undressing. |
