# Supplementary material for: Development and Validation of Daping Orthopedics Operative Risk Scoring System for Geriatric Hip Fracture (DOORS‐GHF) for Predicting Postoperative Outcomes in Geriatric Hip Fracture Patients: A Multicenter Retrospective Comparative Study
Source: Orthop Surg. 2026 Sep 27:10.1111/os.70430. Online ahead of print. doi: 10.1111/os.70430 (PMC13616831; doi:10.1111/os.70430)
Supplement: Supplementary file 1 [file OS-9999-0-s002.docx]

**SUPPLEMENTARY TABLE 3 | Diabetes Classification.**

| **Health Status Grade** | **Physical and Functional Status of Older Patients with Diabetes** |
| --- | --- |
| Group 1 | non-diabetes |
| Group 2 | No comorbidity or has ≤2 non-diabetes chronic illnesses (including stroke, hypertension, stage 1–3 chronic kidney disease, osteoarthritis, etc.) and no ADL impairment and ≤1 IADL impairment |
| Group 3 | Three or more non-diabetes chronic illnesses (including stroke, hypertension, stage 1–3 chronic kidney disease, osteoarthritis, etc.) and/or any one of the following: (1) mild cognitive impairment or early dementia; (2) ≥2 IADL impairments |
| Group 4 | Any one of the following: (1) one or more chronic illnesses with limited treatments and reduced life expectancy (including metastatic malignancies, lung disease requiring oxygen therapy, end-stage renal disease requiring dialysis, and advanced heart failure); (2) moderate to severe dementia; (3) ≥2 ADL impairments; (4) residence in a long-term nursing facility |

Note: ADL = Activities of Daily Living; IADL = Instrumental Activities of Daily Living.
