# Supplementary material for: Development and Validation of Daping Orthopedics Operative Risk Scoring System for Geriatric Hip Fracture (DOORS‐GHF) for Predicting Postoperative Outcomes in Geriatric Hip Fracture Patients: A Multicenter Retrospective Comparative Study
Source: Orthop Surg. 2026 Sep 27:10.1111/os.70430. Online ahead of print. doi: 10.1111/os.70430 (PMC13616831; doi:10.1111/os.70430)
Supplement: Supplementary file 2 [file OS-9999-0-s004.docx]

**SUPPLEMENTARY TABLE 1 | Baseline Characteristics Comparison: Complete cohort (N = 1,599) vs. Imputation cohort (N = 630).**

| **Parameters** | **Complete cohort (N = 1,599)** | **Imputation cohort (N = 630)** | **Statistics** | ***P* value** |
| --- | --- | --- | --- | --- |
| **Gender, n (%)** |  |  | **χ2 = 0.000** | 0.986 |
| male | 460 (28.768) | 181 (28.730) |  |  |
| female | 1139 (71.232) | 449 (71.270) |  |  |
| **Age, (year), M (Q₁, Q₃)** | **80.000**  **(73.500, 85.000)** | **79.000**  **(73.000, 84.000)** | **Z = 1.513** | 0.130 |
| **Cardiac function Classification, n(%)** |  |  | **Z = 4.601** | **<0.001** |
| I | 115 (7.192) | 150 (23.810) |  |  |
| II | 704 (44.028) | 189 (30.000) |  |  |
| III | 449 (28.080) | 180 (28.571) |  |  |
| IV | 331 (20.700) | 111 (17.619) |  |  |
| **WBC, M (Q₁, Q₃)** | **7.560 (6.120, 9.330)** | **7.505 (6.133, 9.518)** | **Z = -0.489** | 0.625 |
| **HB, Mean±SD** | **116.194±19.015** | **118.259±17.605** | **t = -2.356** | **0.019** |
| **BUN, M (Q₁, Q₃)** | **6.200 (4.900, 7.790)** | **6.100 (4.883, 7.540)** | **Z = 1.439** | 0.15 |
| **Systolic pressure (mmHg), Mean±SD** | **140.345±20.800** | **139.633±19.622** | **t = 0.739** | 0.46 |
| **Fracture type (Garden/Evans), n (%)** |  |  | **χ2 = 6.509** | 0.59 |
| Garden | 1168 (73.046) | 485 (76.984) |  |  |
| Evans | 431 (26.954) | 145 (23.016) |  |  |
| **Hypertension, n(%)** |  |  | **χ² = 4.422** | **0.035** |
| No | 899 (56.223) | 385 (61.111) |  |  |
| Yes | 700 (43.777) | 245 (38.889) |  |  |
| **Cancer, n (%)** |  |  | **χ² = 0.696** | 0.404 |
| No | 1542 (96.435) | 612 (97.143) |  |  |
| Yes | 57 (3.565) | 18 (2.857) |  |  |
| **Coronary heart disease, n (%)** |  |  | **χ² = 3.192** | 0.074 |
| No | 1159 (72.483) | 480 (76.190) |  |  |
| Yes | 440 (27.517) | 150 (23.810) |  |  |
| **Cerebrovascular disease, n (%)** |  |  | **χ² = 0.370** | 0.543 |
| No | 1349 (84.365) | 538 (85.397) |  |  |
| Yes | 250 (15.635) | 92 (14.603) |  |  |
| **Nervous system disease, n (%)** |  |  | **χ² = 0.037** | 0.848 |
| No | 1463 (91.495) | 578 (91.746) |  |  |
| Yes | 136 (8.505) | 52 (8.254) |  |  |
| **Respiratory system disease, n (%)** |  |  | **χ² = 5.528** | **0.019** |
| No | 1329 (83.114) | 549 (87.143) |  |  |
| Yes | 270 (16.886) | 81 (12.857) |  |  |
| **Digestive system disease, n (%)** |  |  | **χ² = 5.163** | **0.023** |
| No | 1471 (91.995) | 597 (94.762) |  |  |
| Yes | 128 (8.005) | 33 (5.238) |  |  |
| **Hepatic/Renal insufficiency, n (%)** |  |  | **χ² = 16.518** | **<0.001** |
| No | 1451 (90.744) | 604 (95.873) |  |  |
| Yes | 148 (9.256) | 26 (4.127) |  |  |
| **Diabetes, n (%)** |  |  | **χ² = 7.349** | **0.007** |
| No | 1256 (78.549) | 527 (83.651) |  |  |
| Yes | 343 (21.451) | 103 (16.349) |  |  |
| **Ectopic rhythm, n (%)** |  |  | **χ² = 0.632** | 0.427 |
| No | 1466 (91.682) | 584 (92.698) |  |  |
| Yes | 133 (8.318) | 46 (7.302) |  |  |
| **Tachyarrhythmia, n (%)** |  |  | **χ² = 0.093** | 0.761 |
| No | 1471 (91.995) | 582 (92.381) |  |  |
| Yes | 128 (8.005) | 48 (7.619) |  |  |
| **Conduction disorder, n (%)** |  |  | **χ2 = 0.9911** | 0.911 |
| No | 1392 (87.055) | 541 (85.873) |  |  |
| Atrial Premature Beats | 136 (8.505) | 59 (9.365) |  |  |
| Supraventricular Premature Contractions | 46 (2.877) | 21 (3.333) |  |  |
| Ventricular Premature Contractions | 25 (1.563) | 9 (1.429) |  |  |
| **Premature beat, n (%)** |  |  | **χ2 = 1.339** | 0.855 |
| No | 1324 (82.802) | 530 (84.127) |  |  |
| Yes | 275 (17.198) | 100 (15.873) |  |  |
| **ECG band change, n (%)** |  |  | **χ² = 0.033** | 0.855 |
| No | 1289 (80.613) | 510 (80.952) |  |  |
| Yes | 310 (19.387) | 120 (19.048) |  |  |
| **Operation time (min), M (Q₁, Q₃)** | **80.000**  **(65.000, 105.000)** | **80.000**  **(65.000, 108.750)** | **Z = -0.081** | 0.936 |
| **Bleeding volume (mL), n (%)** |  |  | **Z = 0.821** | 0.411 |
| 50 | 11 (0.688) | 4 (0.635) |  |  |
| 100 | 69 (4.315) | 36 (5.714) |  |  |
| 150 | 86 (5.378) | 31 (4.921) |  |  |
| 200 | 270 (16.886) | 121 (19.206) |  |  |
| 250 | 118 (7.380) | 44 (6.984) |  |  |
| 300 | 289 (18.074) | 107 (16.984) |  |  |
| 350 | 143 (8.943) | 45 (7.143) |  |  |
| 400 | 232 (14.509) | 90 (14.286) |  |  |
| 450 | 120 (7.505) | 48 (7.619) |  |  |
| ≥500 | 261 (16.323) | 104 (16.508) |  |  |

Note: M: median; Q₁: 25th percentile; Q₃: 75th percentile; SD: standard deviation; WBC: white blood cell; HB: hemoglobin; BUN: blood urea nitrogen; ECG: electrocardiogram.
