# Supplementary material for: Development and Validation of Daping Orthopedics Operative Risk Scoring System for Geriatric Hip Fracture (DOORS‐GHF) for Predicting Postoperative Outcomes in Geriatric Hip Fracture Patients: A Multicenter Retrospective Comparative Study
Source: Orthop Surg. 2026 Sep 27:10.1111/os.70430. Online ahead of print. doi: 10.1111/os.70430 (PMC13616831; doi:10.1111/os.70430)
Supplement: Supplementary file 3 [file OS-9999-0-s005.docx]

**SUPPLEMENTARY TABLE 4 | Cardiac Function Classification.**

| **Cardiac Function Classification** | **Specific Contents of Cardiac Function Classification** |
| --- | --- |
| A (Class I) | No history of heart disease; or history of heart disease but no limitation of ordinary physical activity. Breath-holding time > 30 seconds. |
| B (Class II) | History of heart disease with slight limitation of ordinary physical activity. Breath-holding time 20–30 seconds. |
| C (Class III) | History of heart disease with marked limitation of ordinary physical activity. Breath-holding time 10–20 seconds. |
| D (Class IV) | History of heart disease with severe limitation of ordinary physical activity, unable to carry on any physical activity without discomfort. Breath-holding time < 10 seconds or unable to complete the test. |
