# Supplementary material for: Development and Validation of Daping Orthopedics Operative Risk Scoring System for Geriatric Hip Fracture (DOORS‐GHF) for Predicting Postoperative Outcomes in Geriatric Hip Fracture Patients: A Multicenter Retrospective Comparative Study
Source: Orthop Surg. 2026 Sep 27:10.1111/os.70430. Online ahead of print. doi: 10.1111/os.70430 (PMC13616831; doi:10.1111/os.70430)
Supplement: Supplementary file 6 [file OS-9999-0-s009.docx]

**SUPPLEMENTARY TABLE 6 | Surgical Grading.**

| **Classification of surgery** | **Official Definition Description** |
| --- | --- |
| Minor | Low risk, simple procedure, low technical difficulty. |
| Moderate | Moderate risk, moderately complex procedure, moderate technical difficulty. |
| Major | High risk, relatively complex procedure, high technical difficulty. |
| Extra-major | High risk, complex procedure, great technical difficulty; major surgery. |
