# Supplementary material for: Development and Validation of Daping Orthopedics Operative Risk Scoring System for Geriatric Hip Fracture (DOORS‐GHF) for Predicting Postoperative Outcomes in Geriatric Hip Fracture Patients: A Multicenter Retrospective Comparative Study
Source: Orthop Surg. 2026 Sep 27:10.1111/os.70430. Online ahead of print. doi: 10.1111/os.70430 (PMC13616831; doi:10.1111/os.70430)
Supplement: Supplementary file 7 [file OS-9999-0-s010.docx]

**SUPPLEMENTARY TABLE 8 | Estimated Intraoperative Blood Loss by Surgical Procedure and Age Group.**

| **Age Group** | **Hemiarthroplasty** | | | | **Total Hip Arthroplasty** | | | |
| --- | --- | --- | --- | --- | --- | --- | --- | --- |
|  | Number of cases (n) | Mean blood loss (mL) | Standard deviation (SD) | Mean ± SD (mL) | Number of cases (n) | Mean blood loss (mL) | Standard deviation (SD) | Mean ± SD (mL) |
| 65 years | 4 | 250 | 147.2 | 250.0±147.2 | 26 | 363.5 | 115.4 | 363.5±115.4 |
| 66 years | 7 | 257.1 | 123.9 | 257.1±123.9 | 19 | 368.4 | 146.4 | 368.4±146.4 |
| 67 years | 1 | 400 | - | 400 | 15 | 390 | 150.2 | 390.0±150.2 |
| 68 years | 7 | 285.7 | 124.9 | 285.7±124.9 | 21 | 381 | 128.9 | 381.0±128.9 |
| 69 years | 6 | 308.3 | 135.7 | 308.3±135.7 | 17 | 411.8 | 132.9 | 411.8±132.9 |
| 70 years | 5 | 300 | 145.8 | 300.0±145.8 | 20 | 422.5 | 136.2 | 422.5±136.2 |
| 71 years | 8 | 231.2 | 103.3 | 231.2±103.3 | 33 | 404.5 | 156.3 | 404.5±156.3 |
| 72 years | 16 | 293.8 | 143.6 | 293.8±143.6 | 17 | 435.3 | 127.2 | 435.3±127.2 |
| 73 years | 10 | 315 | 147.3 | 315.0±147.3 | 22 | 409.1 | 174.3 | 409.1±174.3 |
| 74 years | 10 | 295 | 79.8 | 295.0±79.8 | 18 | 436.1 | 137 | 436.1±137.0 |
| 75 years | 20 | 315 | 143.4 | 315.0±143.4 | 27 | 455.6 | 129.6 | 455.6±129.6 |
| 76 years | 17 | 300 | 134.6 | 300.0±134.6 | 30 | 416.7 | 140.4 | 416.7±140.4 |
| 77 years | 12 | 283.3 | 124.9 | 283.3±124.9 | 17 | 370.6 | 114.6 | 370.6±114.6 |
| 78 years | 24 | 264.6 | 98.3 | 264.6±98.3 | 27 | 422.2 | 154.6 | 422.2±154.6 |
| 79 years | 32 | 267.2 | 126.8 | 267.2±126.8 | 25 | 422 | 116.4 | 422.0±116.4 |
| 80 years | 34 | 288.2 | 106.6 | 288.2±106.6 | 30 | 405 | 145.2 | 405.0±145.2 |
| 81 years | 46 | 269.6 | 111.3 | 269.6±111.3 | 16 | 490.6 | 162.5 | 490.6±162.5 |
| 82 years | 40 | 296.2 | 111.7 | 296.2±111.7 | 22 | 402.3 | 110.7 | 402.3±110.7 |
| 83 years | 40 | 286.2 | 88.4 | 286.2±88.4 | 18 | 438.9 | 147.1 | 438.9±147.1 |
| 84 years | 50 | 251 | 96.6 | 251.0±96.6 | 17 | 417.6 | 119.8 | 417.6±119.8 |
| 85 years | 44 | 273.9 | 104.3 | 273.9±104.3 | 17 | 452.9 | 141.9 | 452.9±141.9 |
| 86 years | 38 | 286.8 | 122.9 | 286.8±122.9 | 15 | 440 | 121.3 | 440.0±121.3 |
| 87 years | 42 | 277.4 | 107.2 | 277.4±107.2 | 15 | 436.7 | 115.7 | 436.7±115.7 |
| 88 years | 31 | 272.6 | 99.9 | 272.6±99.9 | 8 | 437.5 | 106.1 | 437.5±106.1 |
| 89 years | 28 | 305.4 | 121.2 | 305.4±121.2 | 14 | 396.4 | 99 | 396.4±99.0 |
| ≥90 years | 98 | 281.1 | 109.9 | 281.1±109.9 | 23 | 482.6 | 129.3 | 482.6±129.3 |
